# Screening Anionic Groups Within Zwitterionic Additives for Eliminating Hydrogen Evolution and Dendrites in Aqueous Zinc Ion Batteries

**DOI:** 10.1007/s40820-025-01826-w

**Published:** 2025-06-26

**Authors:** Biao Wang, Chaohong Guan, Qing Zhou, Yiqing Wang, Yutong Zhu, Haifeng Bian, Zhou Chen, Shuangbin Zhang, Xiao Tan, Bin Luo, Shaochun Tang, Xiangkang Meng, Cheng Zhang

**Affiliations:** 1 Australian Institute for Bioengineering and Nanotechnology, The University of Queensland, Queensland, 4072 Australia; 2 Collaborative Innovation Center of Advanced Microstructures, Jiangsu Key Laboratory of Artificial Functional Materials, College of Engineering and Applied Sciences, National Laboratory of Solid State Microstructures, Nanjing University, Nanjing, 210093 People’s Republic of China; 3 University of Michigan-Shanghai Jiao Tong University Joint Institute, Shanghai Jiao Tong University, Shanghai, 200240 People’s Republic of China

**Keywords:** Zwitterions, Electrolyte additives, Zinc deposition, Aqueous batteries

## Abstract

Zwitterionic materials with covalently tethered cations and anions have great potential as electrolyte additives for aqueous Zn-ion batteries (AZIBs) owing to their appealing intrinsic characteristics and merits. However, the impact of cationic and anionic moieties within zwitterions on enhancing the performance of AZIBs remains poorly understood. Herein, three zwitterions, namely carboxybetaine methacrylate (CBMA), sulfobetaine methacrylate (SBMA), and 2-methacryloyloxyethyl phosphorylcholine (MPC), were selected as additives to investigate their different action mechanisms in AZIBs. All three zwitterions have the same quaternary ammonium as the positively charged group, but having different negatively charged segments, i.e., carboxylate, sulfonate, and phosphate for CBMA, SBMA, and MPC, respectively. By systematical electrochemical analysis, these zwitterions all contribute to enhanced cycling life of Zn anode, with MPC having the most pronounced effect, which can be attributed to the synergistic effect of positively quaternary ammonium group and unique negatively phosphate groups. As a result, the Zn//Zn cell with MPC as additive in ZnSO_4_ electrolyte exhibits an ultralong lifespan over 5000 h. This work proposes new insights to the future development of multifunctional zwitterionic additives for remarkably stable AZIBs.

**Graphical Abstract:**

**Supplementary Information:**

The online version contains supplementary material available at 10.1007/s40820-025-01826-w.

## Highlights

- Three zwitterions with identical quaternary ammonium cations but distinct anionic groups, i.e. carboxylate, sulfonate and phosphate, show distinct impact in aqueous zinc ion batteries.
- The zwitterion with phosphate group (2-methacryloyloxyethyl phosphorylcholine, MPC) uniquely promotes oriented Zn (002) plane deposition and provides pH buffering capability, effectively suppressing dendrite formation and side reactions.
- The Zn//NaVO full cell with MPC additive delivers an ultralong cycling life of 4000 cycles with an exceptionally low capacity decay of 0.014% per cycle.

**Supplementary Information:**

The online version contains supplementary material available at 10.1007/s40820-025-01826-w.

## Graphical abstract

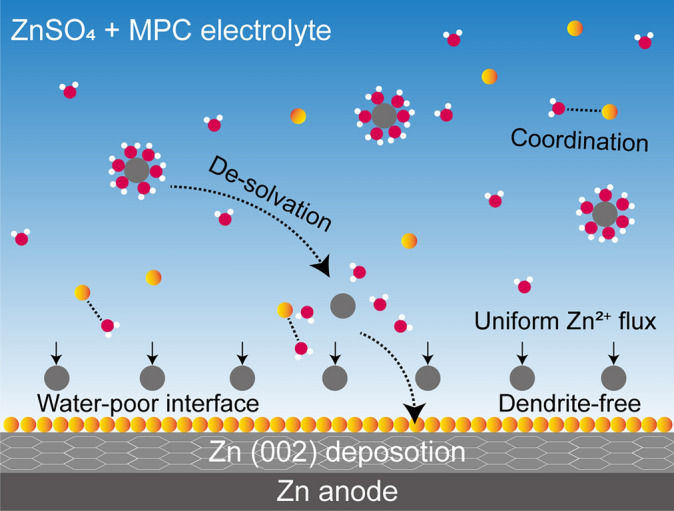

## Introduction

Given the abundant resources and high theoretical capacity (820 mAh g^−1^) of Zn, aqueous Zn ion batteries (AZIBs) have garnered significant attention for large-scale energy storage [1–4]. Unfortunately, the commercialization of AZIBs still suffers from the poor reversibility of Zn anodes during plating/stripping. On the one hand, the tip effect results in an inhomogeneous distribution of Zn^2+^ flux at the electrolyte/electrode interface, inducing uneven Zn deposition [5–7]. Uncontrollable Zn dendrite deposits can increase the risk of internal short circuits. On the other hand, the continuous hydrogen evolution reaction (HER) leads to pH fluctuation engendering the formation of insulated Zn_4_(OH)_6_SO_4_·xH_2_O by-products [8–10]. Importantly, these side reactions and by-products aggravate the irregularity of the electric field and Zn^2+^ flux at the interface, further causing disordered Zn dendrites and providing more reaction sites for undesired HER [11].

Up to now, significant endeavors, including Zn anode modification, artificial interface layers, separator designing and electrolyte optimization, have been adopted to circumvent above issues [12–15]. Among these strategies, electrolyte optimization has been widely employed as an effective and practical solution to stabilize Zn anodes. Typically, functional additives for AZIBs can be divided into two types based on their different functions. Parts of those were used to adsorb onto the Zn surface and contribute to uniform zinc ion transportation [16–18]. The other parts were employed to optimize the solvation structure of Zn^2+^, thereby inhibiting the long-lasting detrimental water molecule-induced parasitic reactions [19–21]. Although some progress has been made, there are still several key issues: (1) single-functional additives are insufficient to simultaneously regulate the deposition behavior of Zn^2+^ and optimize the solvation structure of hydrated Zn^2+^ at the same time; (2) insoluble organic additives rely on the use of expensive organic Zn salts to improve the solubility in H_2_O, which seriously increases the cost; (3) some additives even have a certain toxicity, leading to serious environmental and economic problems.

Zwitterions with covalently tethered cations and anions are non-toxic organic salts that have been used in various fields such as biomedical materials, sensors, and wastewater recycling [22]. Common cations are quaternary ammonium cations, while carboxylate, sulfonate, and phosphate have been used as the counter anions. Recently, owing to their strong hydration ability, zwitterions also show great potential in AZIBs as electrolyte additives [23–27]. For example, Zha et al*.* exploited zwitterionic trifluoro ((4-methyl-morpholino-4-ium) methyl) borate as a novel solid electrolyte interphase (SEI)-forming additive for suppressing H_2_O-induced parasitic reactions [28]. Liu et al*.* demonstrated that zwitterion can be absorbed onto the surface of Zn by –SO_3_^−^ group to protect the Zn anode from electrolyte [29]. Yu et al*.* reported that the carboxylate group provides a prominent interaction with Zn^2+^ to regulate uniform Zn^2+^ flux on the whole electrode [30]. Although some studies have optimized the stability of Zn anode to some extent via zwitterionic materials, efforts have been rather disjointed, lacking systematic investigation on the relationship between different cationic and anionic pairs, stability of anode, and battery performance.

In this work, carboxybetaine methacrylate (CBMA), sulfobetaine methacrylate (SBMA), and 2-methacryloyloxyethyl phosphorylcholine (MPC) with similar molecular structures and good biocompatibility were selected as zwitterionic additives for 2 M ZnSO_4_ (ZSO) electrolyte. Due to their good Zn affinity, zwitterions would serve as zincophilic sites and Zn^2+^ migration sites to homogenize the Zn^2+^ distribution over the surface of Zn and effectively guide Zn deposition. Therefore, all three additives enhance the cycling life of Zn anode. On this basis, however, only MPC further induces the Zn (002) deposition and satisfy the requirement of pH buffering due to quaternary ammonium–phosphate pairs, which distinguishes MPC from SBMA and CBMA, enabling a high reversible Zn anode. Consequently, the Zn//Zn cell with ZSO/MPC exhibits an ultralong lifespan of 5000 h at 1 mA cm^−2^/1 mAh cm^−2^. When paired with NaV_3_O_8_·1.5H_2_O (NaVO) cathode, the full cell can steadily run for 4000 cycles at 5 A g^−1^ and withholds excellent discharge capacity of 102.3 mAh g^−1^.

## Experimental Section

### Preparation of Electrolytes

First, ZnSO_4_·7H_2_O was dissolved in deionized (DI) water to prepare 2 M ZnSO_4_ electrolyte. Then, CBMA, SBMA, or MPC was dissolved in the 2 M ZnSO_4_ solution with various mass ratios (*i.e.,* 0.5, 1, 2, 5, and 10 wt%) to prepare additive-containing electrolytes.

### Preparation of NaVO

Typically, 3 g V_2_O_5_ was dispersed into 100 mL 2 M NaCl aqueous solution. After stirring for 96 h at 30 °C, the mixture was washed with methanol and DI water several times and dried by freeze-drying to obtain NaVO. Additional details regarding characterizations, computational methods, and electrochemical tests can be found in Supporting Information.

## Results and Discussion

### Cycling Performance of Zn//Zn and Zn//Cu Cells Using Different Zwitterions

**Fig. 1:**
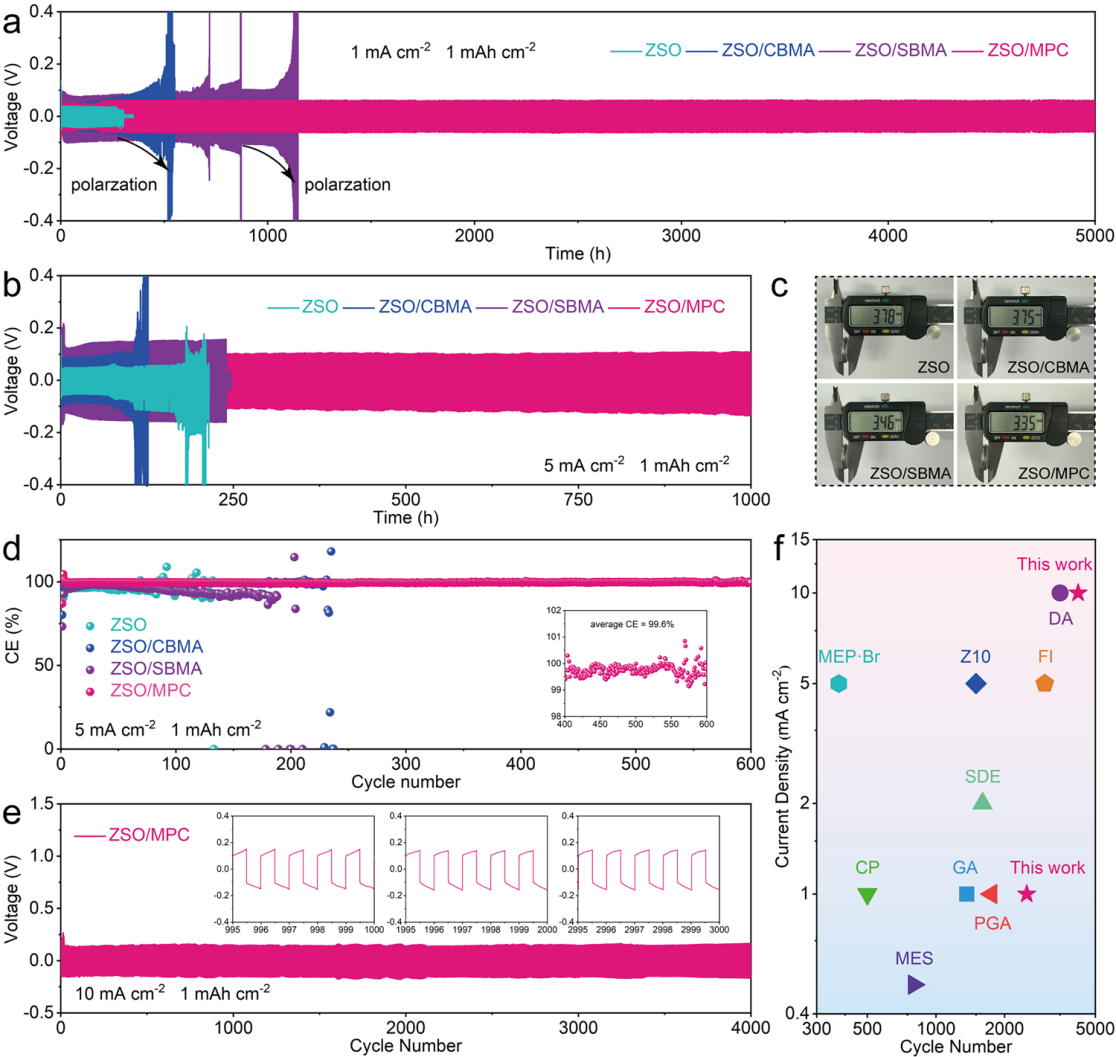
Cycling performance of Zn//Zn cells at **a** 1 mA cm^−2^/1 mAh cm^−2^ and **b** 5 mA cm^−2^/1 mAh cm^−2^. **c** Digital photographs of Zn//Zn cells after cycling. **d** Cycling performance of Zn//Cu cells at 5 mA cm^−2^/1 mAh cm^−2^. **e** Cycling performance of the cell with ZSO/MPC at 10 mA cm^−2^/1 mAh cm^−2^. **f** Performance comparison of MPC with the previously reported electrolyte additives

The plating/stripping performance of Zn electrode is first evaluated by testing symmetric Zn//Zn and asymmetrical Zn//Cu half cells. Before detailed cycling tests, the optimal concentration of additive in ZSO was investigated. According to Figs. S1–S5, low additive concentration cannot provide sufficient protection, while high additive concentration significantly reduces ion conductivity, leading to high polarization voltage. Therefore, the concentration was determined to be 2 wt% for the subsequent characterization and analysis to make comparable results. Figure 1a shows the long-term tests of Zn//Zn cells under typical 1 mA cm^−2^/1 mAh cm^−2^ condition with selected zwitterionic additives (CBMA, SBMA, and MPC). As expected, all zwitterions extend the cycling life of Zn anode. Among them, Zn//Zn cell with ZSO/MPC demonstrates the best stability for 5000 h (Fig. S6), significantly outperforming the cells of ZSO. Unlike the short-circuit failure reflected by a sudden voltage drop in ZSO cell, ZSO/CBMA and ZSO/SBMA cells exhibit a significant increment of overpotential before failure, which is ascribed to the accumulation of passivated products. At 5 mA cm^−2^/1 mAh cm^−2^, the Zn//Zn cell with ZSO/MPC still provides the best performance with stable cycling for 1000 h (Fig. 1b). However, the cells with ZSO/CBMA and ZSO/SBMA show unsatisfactory reversibility. It is worth noting that all cells with ZSO and ZSO/CBMA exhibit significantly voltage fluctuations at both 1 mA cm^−2^/1 mAh cm^−2^ and 5 mA cm^−2^/1 mAh cm^−2^ when approaching battery failure (Figs. S7 and S8). This situation indicates severe HER [31, 32], which can be further demonstrated by post-cycling battery photographs (Fig. 1c). As for ZSO/SBMA cells, Zn dendrites growth at high current density becomes the main challenge.

In addition, the Zn//Cu cell with ZSO/MPC also show superior electrochemical stability of 600 cycles with a high average Coulombic efficiency (CE) of 99.6% (Figs. 1d and S9). In comparison, the CE of ZSO, ZSO/CBMA, and ZSO/SBMA cells suffers from a drastic attenuation within only 140, 230, and 200 cycles, respectively. Impressively, the Zn//Zn cell with ZSO/MPC maintains extraordinary reversibility without significant fluctuations for 4000 cycles at 10 mA cm^−2^/1 mA cm^−2^ and 400 h at an even more aggressive conditions of 10 mA cm^−2^/10 mAh cm^−2^ (Figs. 1e and S10). The unprecedented performance of the ZSO/MPC cell at various conditions exceeds that of most previously reported additives (Fig. 1f and Table S1).

### Surface Characterization of Cycled Zn Anodes

**Fig. 2:**
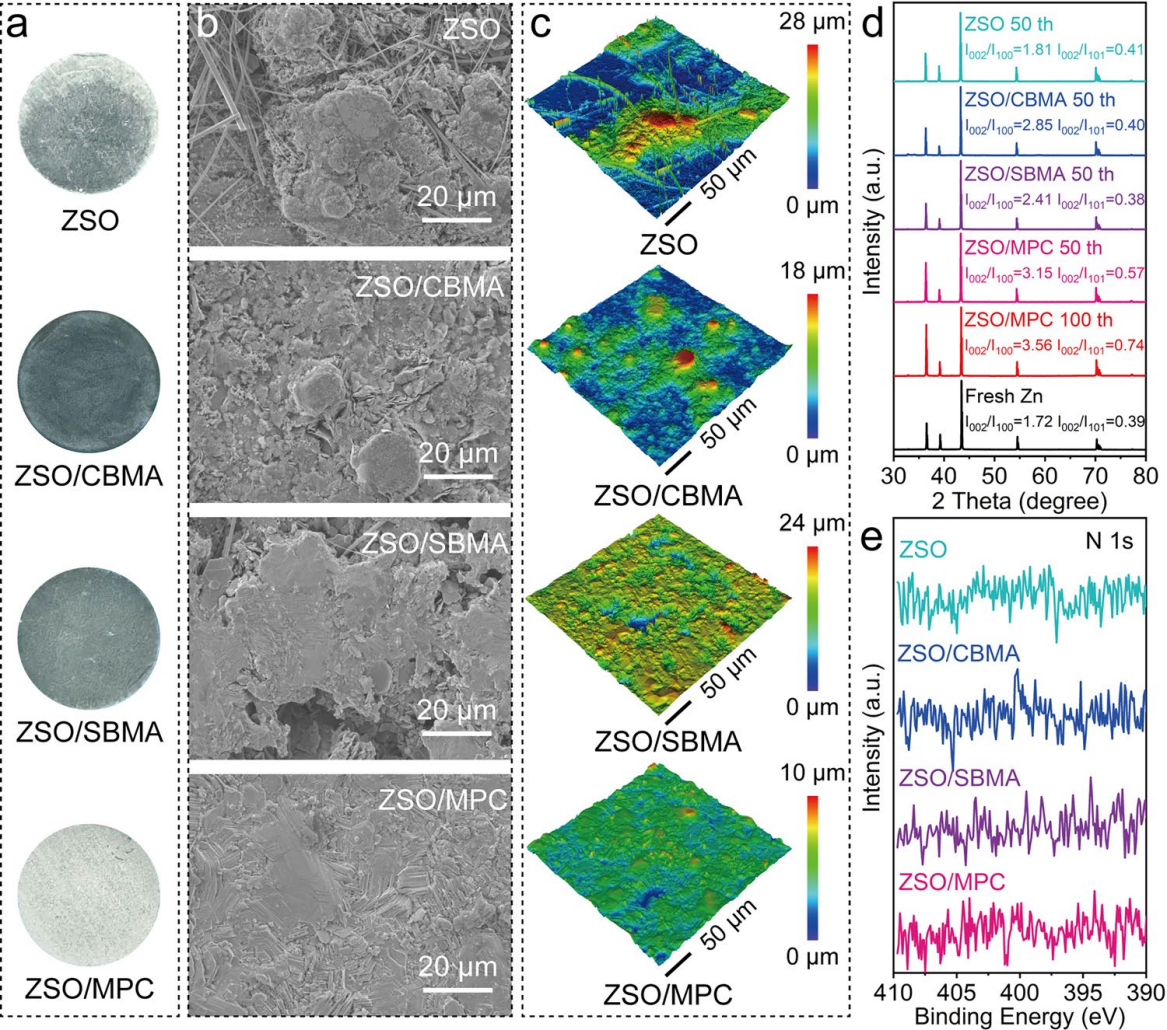
Surface characterization of cycled anode.** a** Digital photographs, **b** SEM images, **c** CLMS height images, **d** XRD patterns, and **e** high-resolution N 1*s* XPS spectra of Zn anode surfaces after cycling in different electrolytes

To confirm the positive effect of zwitterions on Zn anode, we compared the morphology of Zn plates at 1 mA cm^−2^/1 mAh cm^−2^ after 50 cycles. As shown in Fig. 2a, digital photographs first provide a prominent comparison. In the ZSO cell, Zn surface is partially covered with deposited Zn, indicating that Zn plating/stripping is uneven and disordered. In the presence of additives, the whole surface of Zn plates is fully covered with deposited Zn, indicating more complete deposition. Notably, the surface of cycled Zn anode in the ZSO/MPC cell shows a lighter color. Generally, different luster implies different deposition morphologies, which can be revealed by scanning electron microscope (SEM). According to Fig. 2b, the cycled Zn anode in ZSO shows a dendrite-like structure with irregular morphology, which is the primary mechanism for battery failure of upon prolonged cycling. Although CBMA and SBMA alleviate the generation of dendrites and corrosion, Zn anodes still have by-products and holes on the surface due to uneven deposition and corrosion. In comparison, the cycled Zn anode in ZSO/MPC cell shows a homogeneous and tightly packed deposition morphology without dendrite formation and a flat hexagon-tightly packed morphology appeared on the surface (Fig. S11). Similar observations were obtained using confocal laser microscopy (CLMS) images (Figs. 2c and S12). ZSO/MPC cell displays much uniform Zn surface with a much smaller average roughness of 0.84 μm than those of ZSO (5.09 μm), ZSO/CBMA (2.23 μm) and ZSO/SBMA (1.88 μm) cells.

The larger values of I_002_/I_100_ and I_002_/I_101_ usually serve as an indication of uniform and effective Zn deposition due to that the hexagonal Zn (002) has a lower surface energy than the Zn (100) and Zn (101) [33, 34]. As shown in Fig. 2d, the Zn (101) peak exhibits the highest intensity in the initial crystal structure of fresh Zn. After 50 cycles, the significantly higher I_002_/I_100_ and I_002_/I_101_ values for the cycled Zn anode in ZSO/MPC compared to the other electrolytes suggest that MPC has the most pronounced effect. The I_002_/I_100_ and I_002_/I_101_ values of Zn anode after 100 cycles in ZSO/MPC are higher than those of Zn anode after 50 cycles, indicating that MPC can guide the preferential deposition of Zn^2+^ along the (002) crystal plane [12]. In addition, XRD results reveal a significant reduction in the accumulation of Zn_4_(OH)_6_SO_4_·xH_2_O by-products in ZSO/MPC cell (Fig. S13).

Significantly, although these three additives all have quaternary ammonium groups, there are no N 1s X-ray photoelectron spectroscopy (XPS) peaks for cycled Zn anodes in additive-containing electrolytes (Fig. 2e). Furthermore, the elemental analysis of cycled Zn anodes across different electrolytes reveals no significant differences in the content of elements associated with negatively charged groups (C, N, P, and S) (Fig. S14). This suggests that none of these additives undergo decomposition to form a SEI during cycling. Therefore, MPC enables stable Zn anode as an interface regulator, which will be discussed in the later section. As shown in Fig. S15, in situ optical microscope images visually reveal the Zn growth situation at a constant current process. In blank ZSO, irregular dendrites with visible H_2_ bubble are gradually detected in the cross-sectional view of Zn anode with the plating process. In contrast, a dense and uniform deposition layer without H_2_ bubble can be observed in ZSO/MPC cell. The results further support the effectiveness of MPC in inhibiting side reactions and protecting Zn anode.

### Role of Zwitterions in Stabilizing Electrolyte Solution and Interface

**Fig. 3:**
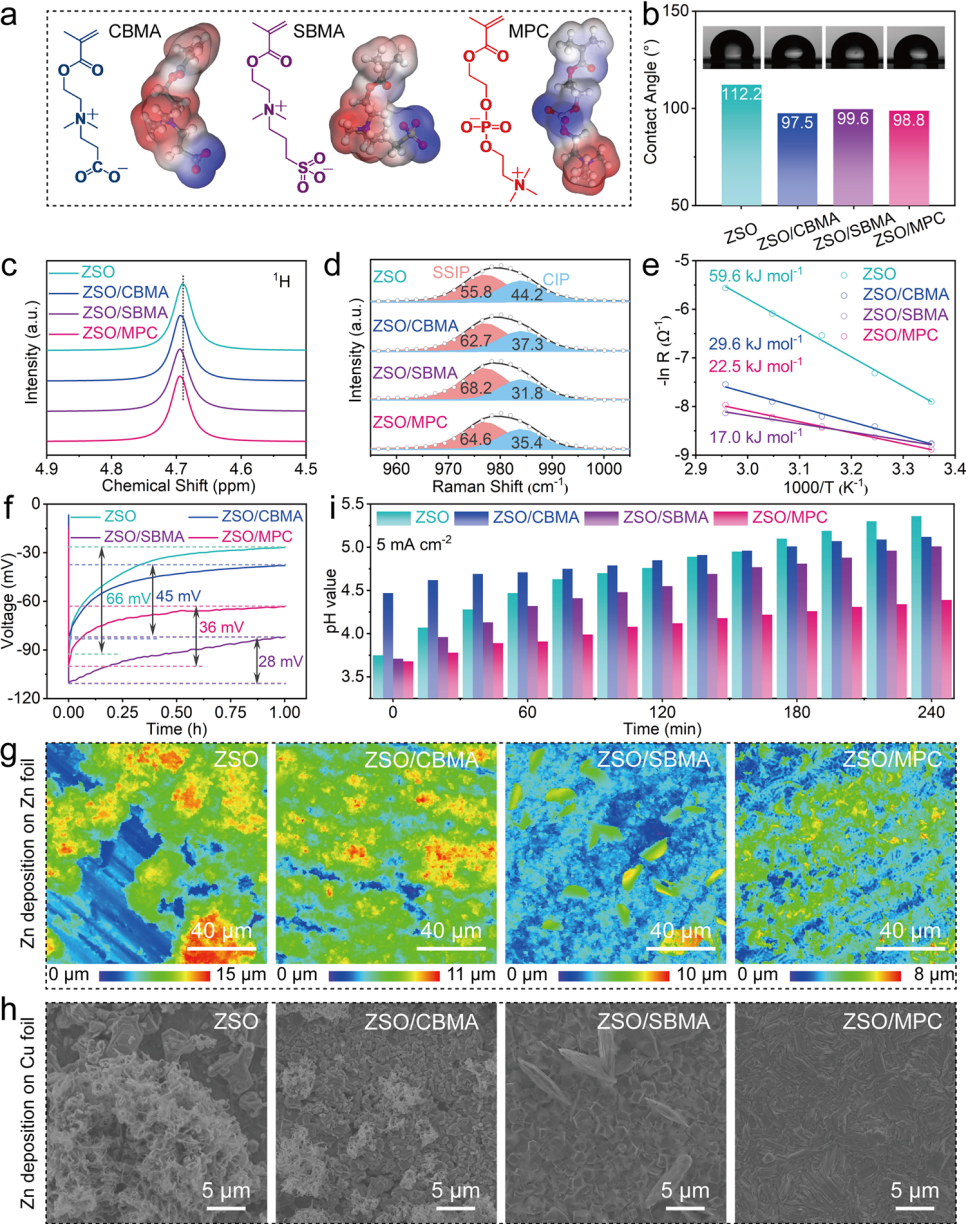
**a** Structural formula and ESP distribution of CBMA, SBMA, and MPC. **b** Wettability tests of different electrolytes on Zn surface. **c** ^1^H NMR and **d** Raman spectra of different electrolytes. **e** Activation energies of different cells. **f** Initial Zn nucleation overpotential of Zn//Zn cells with different electrolytes at 1 mA cm^−2^. **g** CLMS height images of Zn foils and **h** SEM images of Cu foils after plating with Zn at 1 mA cm^−2^/1 mAh cm^−2^. **i** pH change of the different electrolytes during cycling

As shown in Fig. 3a, the zwitterions exhibit more negative electrostatic potential (ESP) values near the negatively charged groups and more positive ESP values near quaternary ammonium groups. The cationic parts of zwitterions preferentially absorb onto the negatively charged anode, while the anionic parts provide a potential Zn^2+^ migration site [24, 35]. In addition, the additive-containing electrolytes remain clear and transparent (Fig. S16), demonstrating the excellent compatibility between these three zwitterions and the ZSO electrolyte. Moreover, the contact angle tests display that zwitterionic additives enhance the wettability between electrolyte and Zn (Fig. 3b), which is beneficial for decreasing the interfacial free energy on the electrode/electrolyte interface [20]. To analyze the effect of different zwitterions on solvated Zn^2+^, nuclear magnetic resonance (NMR) and Raman analysis were conducted. Chemical shifts reflect the local chemical environment surrounding the hydrogen nuclei within a molecule. As shown in Fig. 3c, the ^1^H peaks of additive-containing electrolytes in NMR shift to a higher chemical shift, suggesting reduced electron densities on the relevant ^1^H, suggesting the formation of a strong hydrogen bond between the zwitterions and H_2_O [21, 36]. According to the classic Eigen–Tamm mechanism, the peaks index to SO_4_^2−^ in Raman spectra can be deconvoluted into the contact ion pair (CIP) and the solvent-separated ion pair (SSIP). As shown in Fig. 3d, the proportion of CIP in additive-containing electrolytes are lower than that in ZSO, further indicating that the original solvation structure has been reconstructed due to the addition of zwitterions [37, 38].

Benefiting from some fundamental features of zwitterions, all additives improve the cycling life of Zn anode to a certain degree. Next, we further investigate why different negatively charged groups contribute to varying levels of improvement in cycling performance. Before deposition, the Zn(H_2_O)_6_^2+^ will release H_2_O from their solvated shell. Generally, the active H_2_O molecules derived from hydrated Zn^2+^ are more easily reduced into H_2_ than free H_2_O molecules [19]. Therefore, the fast desolvation is important for HER-suppressed AZIBs. The activation energy (E_a_) of different cells was simulated by Nyquist plots of Zn//Zn cells from 25 to 65 °C (Fig. S17) [31]. As summarized in Fig. 3e, the E_a_ of cells based on ZSO, ZSO/CBMA, ZSO/SBMA, and ZSO/MPC are 45.8, 29.6, 17.0, and 22.5 kJ mol^−1^, respectively, suggesting that Zn(H_2_O)_6_^2+^ desolvation is a key issue in blank ZSO cell. The enhanced desolvation process can reduce the rate of HER, which could be further confirmed by the linear sweep voltammetry (LSV) measurements. As observed in Fig. S18, ZSO/SBMA and ZSO/MPC deliver lower HER potential, revealing the excellent ability of SBMA and MPC in suppressing the water-induced HER (also supported by the results shown in Fig. 1c).

Due to high Zn affinity of zwitterions, the cells with additive-containing electrolytes have lower nucleation energy barriers (Fig. 3f), which is more conducive to uniform Zn nucleation. However, it is worth noting that a low nucleation energy barrier does not necessarily change the crystal plane of deposited Zn. To confirm this speculation, the Zn deposition morphologies on Zn and Cu substrates were further investigated. As shown in Figs. 3g and S19, different electrolytes have significantly different impacts on Zn^2+^ deposition behavior. Guided by the zwitterionic layer covering on Zn, Zn^2+^ prefers to interact with the zincophilic sites of zwitterionic additives, inducing uniform Zn plating. In contrast, large, exposed areas with no deposits exist on cycled Zn anode in ZSO cell. In addition, SEM images further reveal the difference of deposition morphologies in different electrolytes (Figs. 3h and S20). Obviously, there are a large number of mossy-like Zn deposits in ZSO cell due to uneven Zn deposition and parasitic H_2_ evolution. Although the introduction of CBMA and SBMA in ZSO moderately optimizes Zn deposition, little mossy- and sheet-like Zn deposits are still observed in corresponding cells. It is well known that the loose Zn structure will accelerate interfacial side reaction, and the uncontrolled dendrites would cause the short-circuit failure of batteries, in consistent with the half-battery failure mechanism of ZSO/CBMA and ZSO/SBMA cells in Fig. 2.

The different anionic segments of CBMA, SBMA, and MPC exhibit different responses to pH fluctuations during charging/discharging cycles [39, 40]. As shown in Fig. 3i, the pH of blank ZSO electrolyte increases significantly from 3.8 to 5.4, indicating pronounced HER. In contrast, the addition of zwitterion suppresses the changes in pH. To be more specific, the MPC-containing electrolyte maintained a more stable pH compared to those with CBMA and SBMA, indicating its superior pH-buffering capacity. This suggests that the quaternary ammonium group in MPC can associate with OH⁻, while the phosphate group, with multiple ionizable protons (pKa_1_ ≈ 2.1, pKa_2_ ≈ 7.2), is capable of reversibly binding H⁺. Such dual buffering behavior is consistent with the observed improvements in interfacial stability and suppression of side reactions in the MPC system. Therefore, despite being zwitterionic, CBMA and SBMA molecules cannot effectively neutralize H⁺ in mildly acidic environments, nor regulate OH^−^ as efficiently as MPC. These functional differences account for the weaker pH buffering and inferior electrochemical performance observed with CBMA and SBMA additives.

The above results demonstrate that the uneven Zn^2+^ flux, critical side reactions, and uncontrollable Zn deposition in blank ZSO are harmful to Zn anode. After inducing zwitterionic additives, the cationic parts of zwitterions preferentially absorb on the negatively charged anode, while the anionic parts provide a potential Zn^2+^ migration site. Therefore, all three zwitterions show strong interaction with H_2_O and enhanced wettability, which is more conducive to uniform nucleation by regulating Zn^2+^ flux. On this basis, however, only MPC can regulate the preferential Zn (002) crystal plane deposition and satisfy the requirement of pH buffering, thus significantly alleviated dendrites and side reaction at the same time. In contrast, CBMA induces the formation of Zn_4_(OH)_6_SO_4_·xH_2_O by-products and SBMA cannot change the crystal plane of deposited Zn. As previously reported, Zn dendrites, HER, and corrosion are mutually reinforcing and inextricably linked [6, 41]. Therefore, CBMA and SBMA are insufficient to enable a high reversible Zn anode during long-term cycling.

### DFT Calculation and MD Simulation

**Fig. 4:**
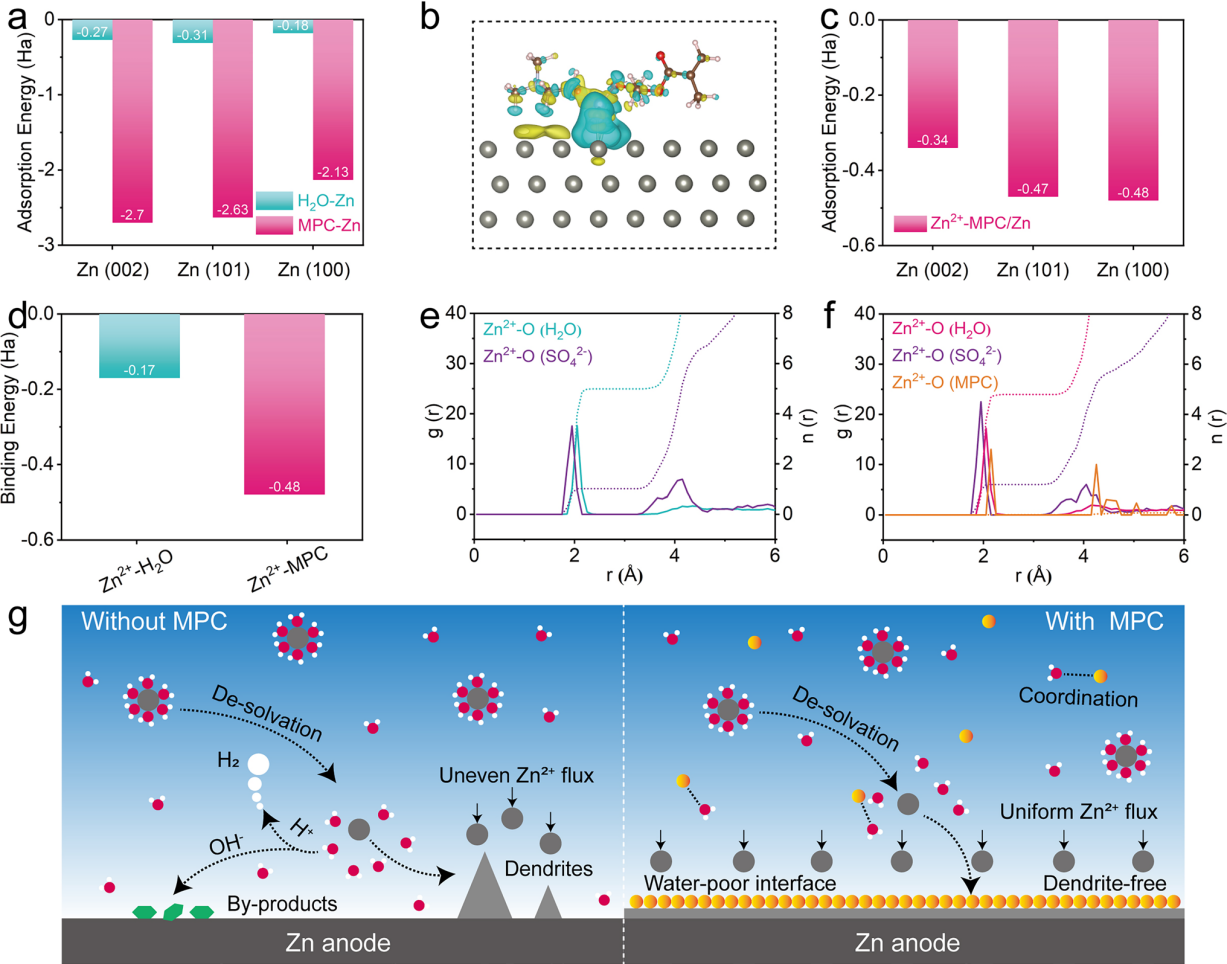
**a** Adsorption energy of H_2_O and MPC on different Zn facets. **b** 3D contour mapping differential charge density of MPC on Zn (002) plane. **c** Adsorption energy of Zn^2+^ on different facets of MPC/Zn. **d** Binding energy between H_2_O, Zn^2+^, and MPC. RDFs and CN of **e** ZSO and **f** ZSO/MPC. **g** Schematic illustrations of the Zn plating behaviors in ZSO and ZSO/MPC

Based on the above analyses, the additives, especially MPC, are believed to have positive effects on Zn anodes. To further analyze and uncover the underlying mechanisms regulating Zn deposition, density functional theory calculation (DFT) and molecular dynamics (MD) simulation were performed. Figure 4a shows that all the adsorption energies (absolute value) of MPC on Zn (002), (101), (100) facets are higher than those of H_2_O on corresponding planes, meaning that MPC more preferentially adsorbs on Zn metal surface, preventing H_2_O molecules from reaching Zn surface. The 3D contour mapping differential charge density of MPC/Zn and H_2_O/Zn models (Figs. 4b and S21) shows that more yellow electron cloud presents on the Zn surface in the MPC/Zn than that in H_2_O/Zn, suggesting strong chemical interaction between MPC and Zn [42]. According to the Bravais law, the crystal plane with the lowest growth rate usually leads to the finally exposed crystal plane. Further calculation of Zn^2+^ adsorption energy on these facets reveals that the energy of Zn^2+^ on MPC/Zn (002) is lower than that on (100) and (101), suggesting a lower growth rate of the (002) orientation. This eventually leads to a greater exposure of (002) facet on its surface (Fig. 4c) [43].

In addition, the binding energy of MPC to Zn^2+^ is obviously higher than that of H_2_O molecular to Zn^2+^ (Fig. 4d), which means that Zn^2+^ is more likely to bind with MPC than H_2_O, thereby affecting the solvated structure of Zn^2+^ [43]. However, the radial distribution functions (RDF) and coordination number (CN) results (Fig. 4e, f) display that the CN of Zn^2+^–O(MPC) is close to 0. This means that MPC affects solvated structure but does not participate in solvation shell. In summary, Fig. 4g illustrates the correlation between the excellent electrochemical properties and the MPC additive. The MPC additive can (i) form a cross-linked hydrogen bond network with H_2_O, (ii) accelerate desolvation, (iii) adsorb on Zn surface to induce uniform Zn nucleation and further Zn (002) deposition, thus achieving hydrogen evolution-suppressed and dendrite-free Zn anode.

### Electrochemical Performance of Full Cells

**Fig. 5:**
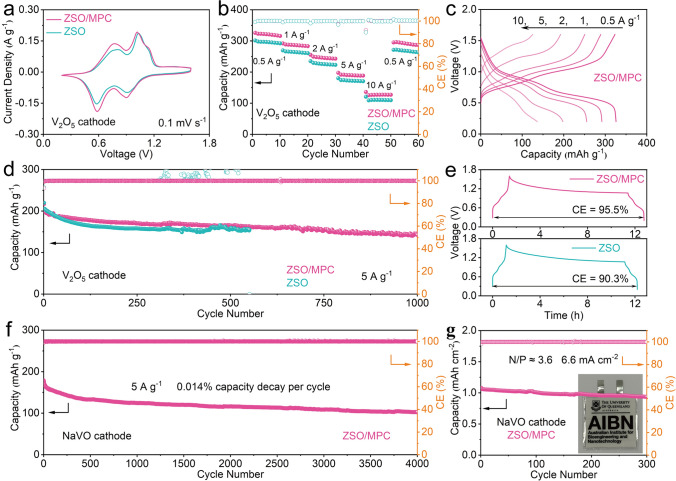
**a** CV curves and **b** rate performance of Zn//V_2_O_5_ cells. **c** GCD curves of Zn//V_2_O_5_ cell with ZSO/MPC at different rates. **d** Cycling performance of Zn//V_2_O_5_ cells at 5 A g^−1^. **e** Self-discharge curves of Zn//V_2_O_5_ cells. **f** Cycling performance of Zn//NaVO cell with ZSO/MPC at 5 A g^−1^. **g** Cycling performance of Zn//NaVO pouch cell with ZSO/MPC at 1 A g^−1^

Following the significantly improved performance of half cells using the MPC additive and a detailed understanding of the enhancement mechanism, we evaluate the performance of full cells using commercial V_2_O_5_ and MnO_2_ (Fig. S22). When paired with V_2_O_5_, the cells with ZSO and ZSO/MPC display similar cyclic voltammetry (CV) peaks (Fig. 5a), suggesting that MPC does not participate in redox reactions during cycling. The cell with the ZSO/MPC shows impressive reversible capacities of 325.9, 291.8, 255.3, 197.7, and 136.0 mAh g^−1^ at 0.5, 1, 2, 5, and 10 A g^−1^, respectively (Fig. 5b). In comparison, specific capacities of the cell with ZSO are lower than those of ZSO/MPC cell at the same rate. Figure 5c presents the galvanostatic charge–discharge (GCD) profiles of ZSO/MPC cell. The charge–discharge plateau is clearly observed even at 10 A g^−1^. Long-term cycling performance further reveals the potential of MPC as a viable additive in practical AZIBs, which remains high-capacity retention of 70.6% after 1,000 cycles (Figs. 5d and S23). However, due to the severe side reactions and uncontrollable Zn dendrites, the ZSO cell shows a sharp capacity drop near 550 cycles. When paired with MnO_2_, the ZSO/MPC cell exhibits improved cycling stability compared to the ZSO cell without additive, highlighting the broad applicability of MPC as an electrolyte additive (Fig. S24). In addition, the ZSO/MPC cell maintains 95.5% capacity after resting of 10 h, which is better than 90.3% of ZSO cell (Fig. 5e).

The good performance was also achieved when using NaVO as the cathode material, which is commonly employed in AZIBs. The morphology and phase characterizations of the as-prepared NaVO are shown in Fig. S25. Attributed to MPC additive, which hinders dendrites and side reactions, the Zn//NaVO cell with ZSO/MPC demonstrates a superior ultralong cycling life over 4000 cycles at 5 A g^−1^ and withholds excellent discharge capacity of 102.3 mAh g^−1^, corresponding to a low capacity decay of 0.014% (Fig. 5f). In addition, Zn//NaVO pouch cells using 20-μm ultra-thin Zn foil as anode were assembled. As shown in Fig. 5g, the pouch cell with ZSO/MPC keeps a capacity retention of 86.6% after 300 cycles at a high current density of 6.6 mA cm^−2^ and a low N/P ≈ 3.6, demonstrating again that MPC is an effective electrolyte additive for AZIBs.

## Conclusions

In conclusion, three zwitterions with same positively charged group (quaternary ammonium) and different negatively charged segments (carboxylate for CBMA, sulfonate for SBMA, and phosphate for MPC) were selected as additives to investigate their different action mechanisms in AZIBs. Benefiting from abundant charged functional groups, all three zwitterions show strong interaction with H_2_O and increased hydrophilicity, which is more conducive to uniform Zn nucleation by regulating Zn^2+^ flux. However, only MPC further regulates the preferential Zn (002) crystal plane deposition and satisfies the requirement of pH buffering, thus significantly alleviated dendrites and side reaction at the same time. This multifunctional effect can be attributed to the synergistic effect of positively quaternary ammonium group and unique negatively phosphate groups. Consequently, the assembled Zn//Zn cell with ZSO/MPC has a cycle life of 4000 cycles at 10 mA cm^−2^/1 mAh cm^−2^. When combined with the NaVO cathode, the Zn//NaVO full cell with ZSO/MPC demonstrates exceptional stability of 4000 cycles with only 0.014% capacity decay per cycle. Additionally, it is important to highlight that MPC includes a methylate group suitable for subsequent polymerization, which allows for straightforward development of more functional polymeric additives and solid electrolytes in future applications.

## Supplementary Information

Below is the link to the electronic supplementary material.

Supplementary file1 (DOCX 23319 KB)
