# Supplementary material for: Screening Anionic Groups Within Zwitterionic Additives for Eliminating Hydrogen Evolution and Dendrites in Aqueous Zinc Ion Batteries
Source: Nanomicro Lett. 2025 Jun 26;17:314. doi: 10.1007/s40820-025-01826-w (PMC12202267; doi:10.1007/s40820-025-01826-w)
Supplement: Supplementary file 1 — Supplementary file1 (DOCX 23319 KB) [file 40820_2025_1826_MOESM1_ESM.docx]

Supporting Information for

**Screening Anionic Groups within Zwitterionic Additives for Eliminating Hydrogen Evolution and Dendrites in Aqueous Zinc Ion Batteries**

Biao Wang^1, 2#^, Chaohong Guan^3#^, Qing Zhou^2#^, Yiqing Wang^1^, Yutong Zhu^1^, Haifeng Bian^2^, Zhou Chen^1^, Shuangbin Zhang^1^, Xiao Tan^1^, Bin Luo^1^, Shaochun Tang^2^*, Xiangkang Meng^2^*, and Cheng Zhang^1^*

^1^ Australian Institute for Bioengineering and Nanotechnology, The University of Queensland, Queensland 4072, Australia

^2^ National Laboratory of Solid State Microstructures, Collaborative Innovation Center of Advanced Microstructures, Jiangsu Key Laboratory of Artificial Functional Materials, College of Engineering and Applied Sciences, Nanjing University, Nanjing 210093, P. R. China

^3^ University of Michigan-Shanghai Jiao Tong University Joint Institute, Shanghai Jiao Tong University, Shanghai 200240, P. R. China

^#^Biao Wang, Chaohong Guan, and Qing Zhou contributed equally to this work.

*Corresponding authors. E-mail: [tangsc@nju.edu.cn](mailto:tangsc@nju.edu.cn) (Shaochun Tang); [mengxk@nju.edu.cn](mailto:mengxk@nju.edu.cn) (Xiangkang Meng); [c.zhang3@uq.edu.au](mailto:c.zhang3@uq.edu.au) (Cheng Zhang)

**S1 Materials characterizations**

The structure and morphology of samples were characterized by the SEM (JEOL JSM-7100F) and CLMS (Leica SP8). XRD (Bruker-D8 ADVANCE) was used to investigate the crystal structures. The elemental status was obtained with XPS (Kratos Axis Supra Plus). Contact angel was measured on a Krűss DSA10 with the sessile drop technique. Raman spectroscopy (WITec alpha300) and NMR (Bruker Avance 500 MHz) were used to investigate the solvated structure of electrolytes.

**S2 Computational methods**

All the density functional theory (DFT) calculations (adsorption energy) were performed by the generalized gradient approximation (GGA) with the exchange-correlation functional PBE. A cutoff energy of 400 eV was used for the plane-wave basis set. The spin geometry optimization conversions with forces on atoms and energy differences were smaller than 0.02 eV Å^-1^ and 10^-5^ eV with a 1×2×1 Monkhorst-Pack k-point mesh, respectively. Moreover, the charge density differences of Zn (002)-H_2_O and Zn (002)/(101)/(100)-MPC were investigated to study the electronic structures.

**S3 Electrochemical tests**

The cathodes were fabricated via mixing active materials, super P, and PVDF at a mass ratio of 7:2:1 in NMP solvent, followed with coating on the Ti foil and drying in a vacuum oven at 60 °C overnight. Electrochemical behaviors were evaluated by using 2032-type coin-cells with glass fiber as the separator. Zn//Zn cells were assembled using Zn foils as the working and counter electrodes. The utilization of Zn at capacities of 1 mAh cm^-2^ and 10 mAh cm^-2^ are approximately 1.7% and 16.7%, respectively. Zn//Cu cells were assembled using Cu foils as the working electrodes and Zn foils as the counter electrodes. Full cells were assembled using Zn foils as anodes with different cathodes. CV tests were carried out at scan rates of 0.1 mV s^-1^ between 0.2 V and 1.6 V on a CHI760E electrochemical workstation. EIS tests were performed on a CHI760E electrochemical workstation with a frequency range of 100 kHz-0.1 Hz.

**S4 Supplementary Figures and Tables**


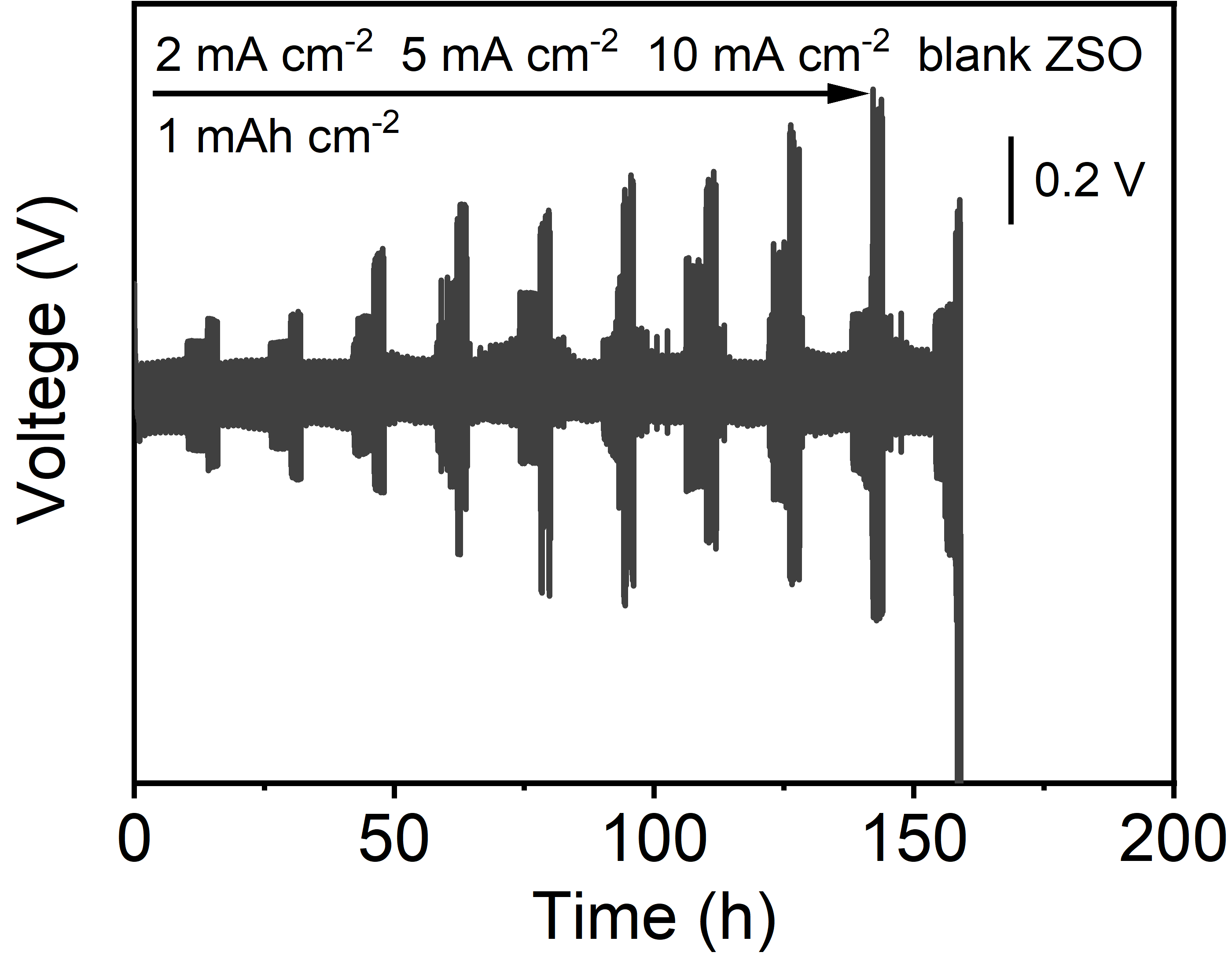


**Fig. S1** Cycling performance of Zn//Zn cell based on blank ZSO electrolyte


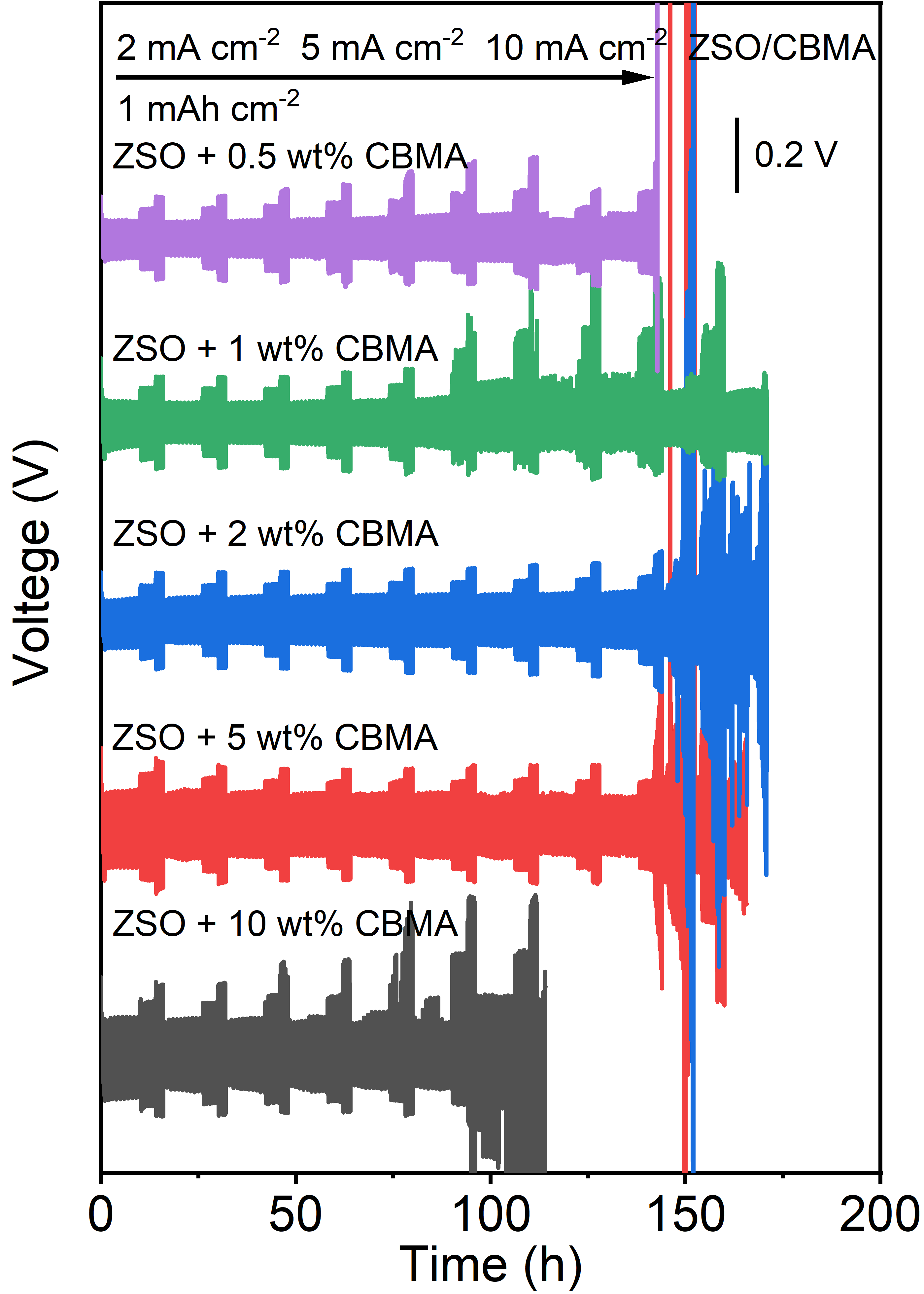


**Fig. S2** Cycling performance of Zn//Zn cells based on ZSO/CBMA electrolytes with the concentration of CBMA from 0.5 to 10 wt%


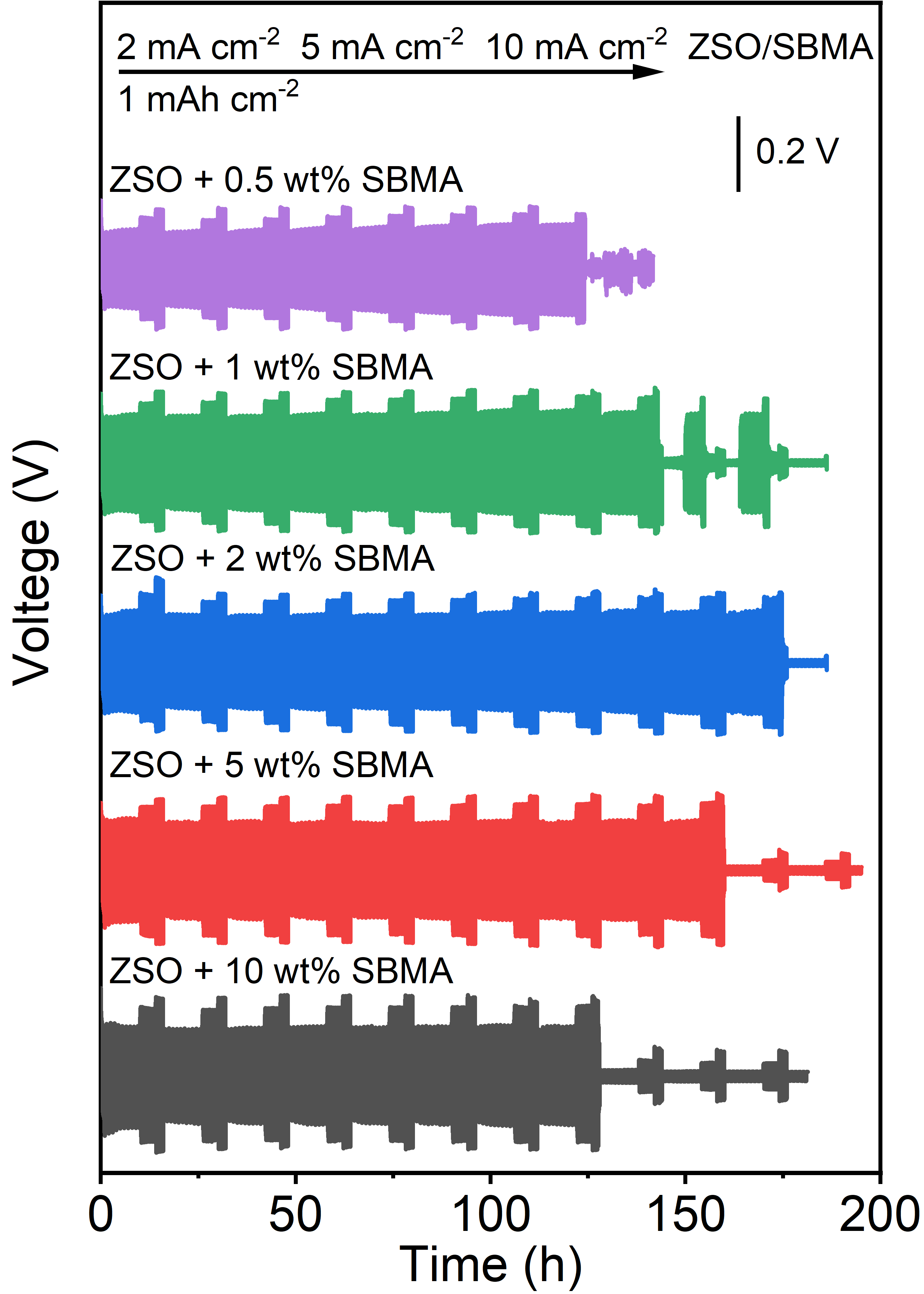


**Fig. S3** Cycling performance of Zn//Zn cells based on ZSO/SBMA electrolytes with the concentration of SBMA from 0.5 to 10 wt%


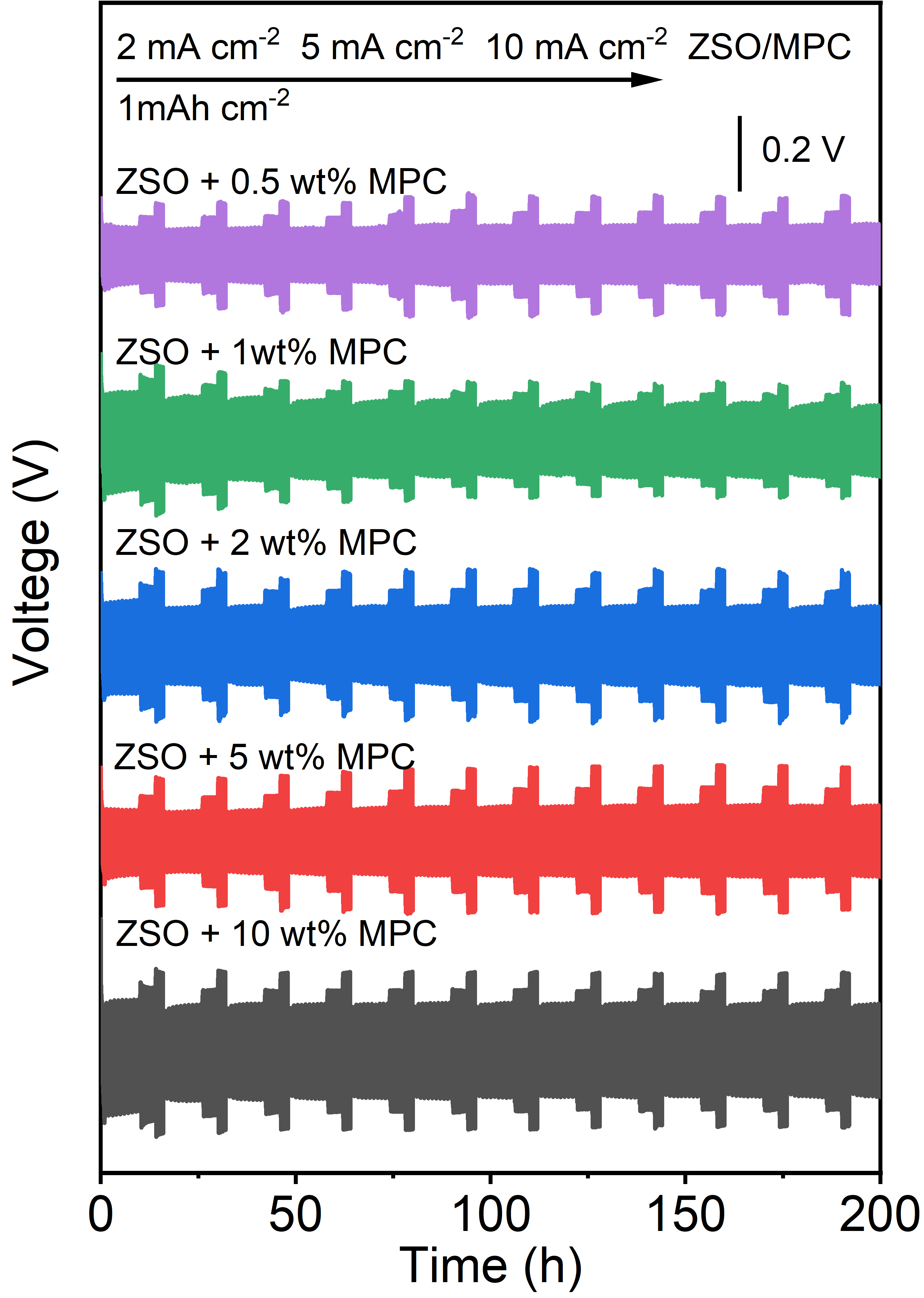


**Fig. S4** Cycling performance of Zn//Zn cells based on ZSO/MPC electrolytes with the concentration of MPC from 0.5 to 10 wt%


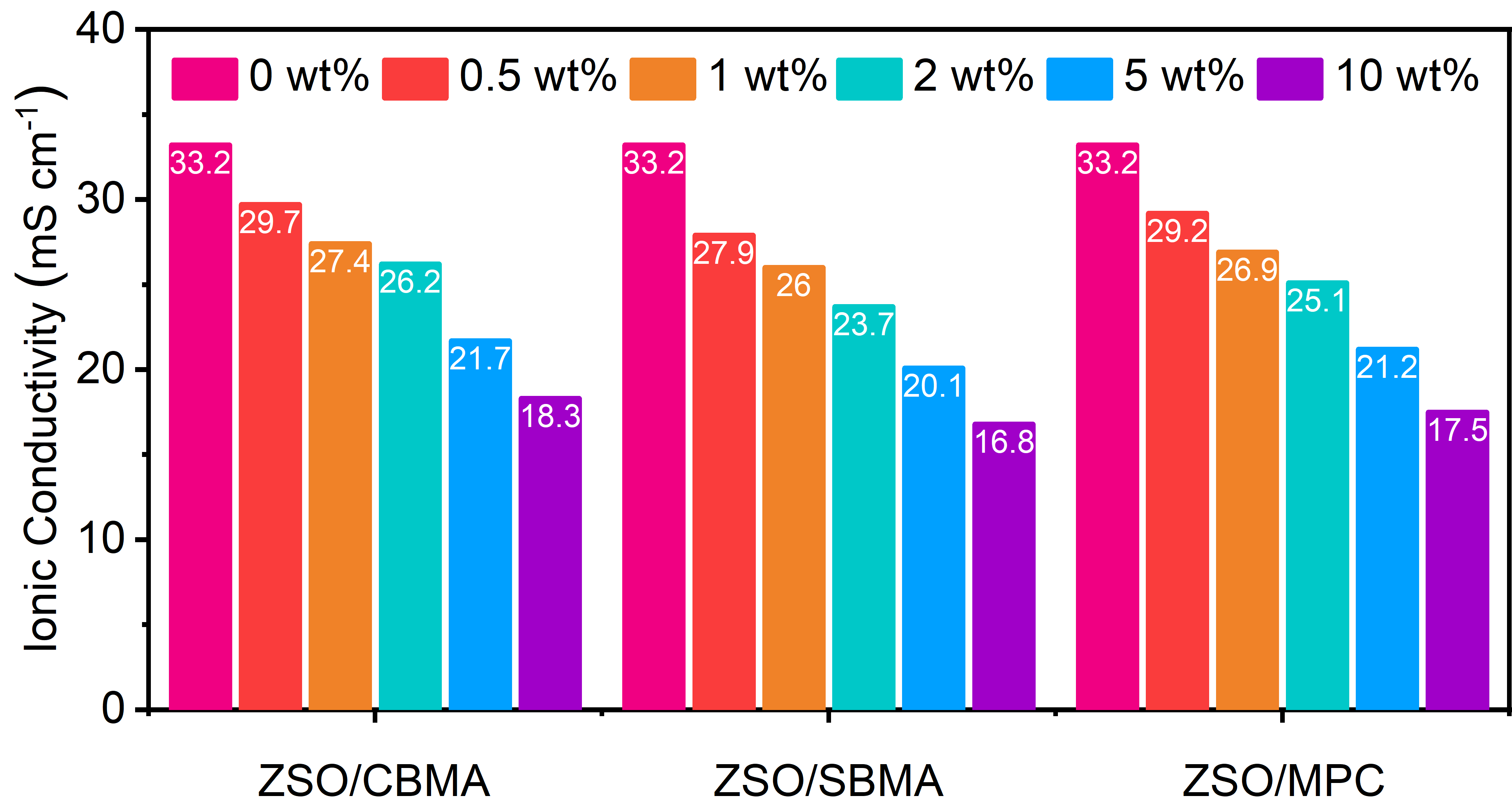


**Fig. S5** Ionic conductivity of different electrolytes
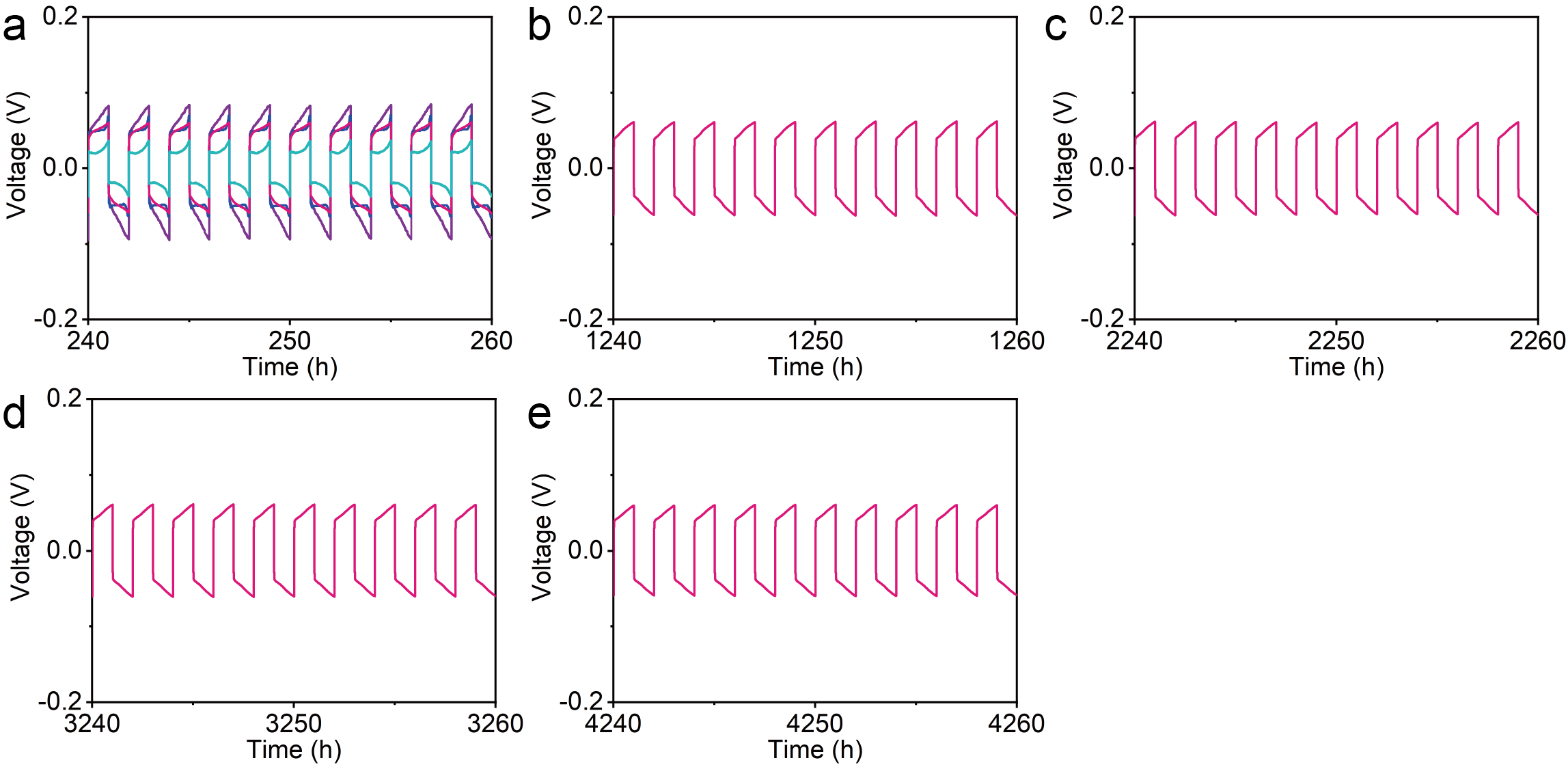


**Fig. S6** Detailed voltage profiles of **Fig. 1a** at about 250 h, 1250 h, 2250 h, 3250 h, and 4250 h


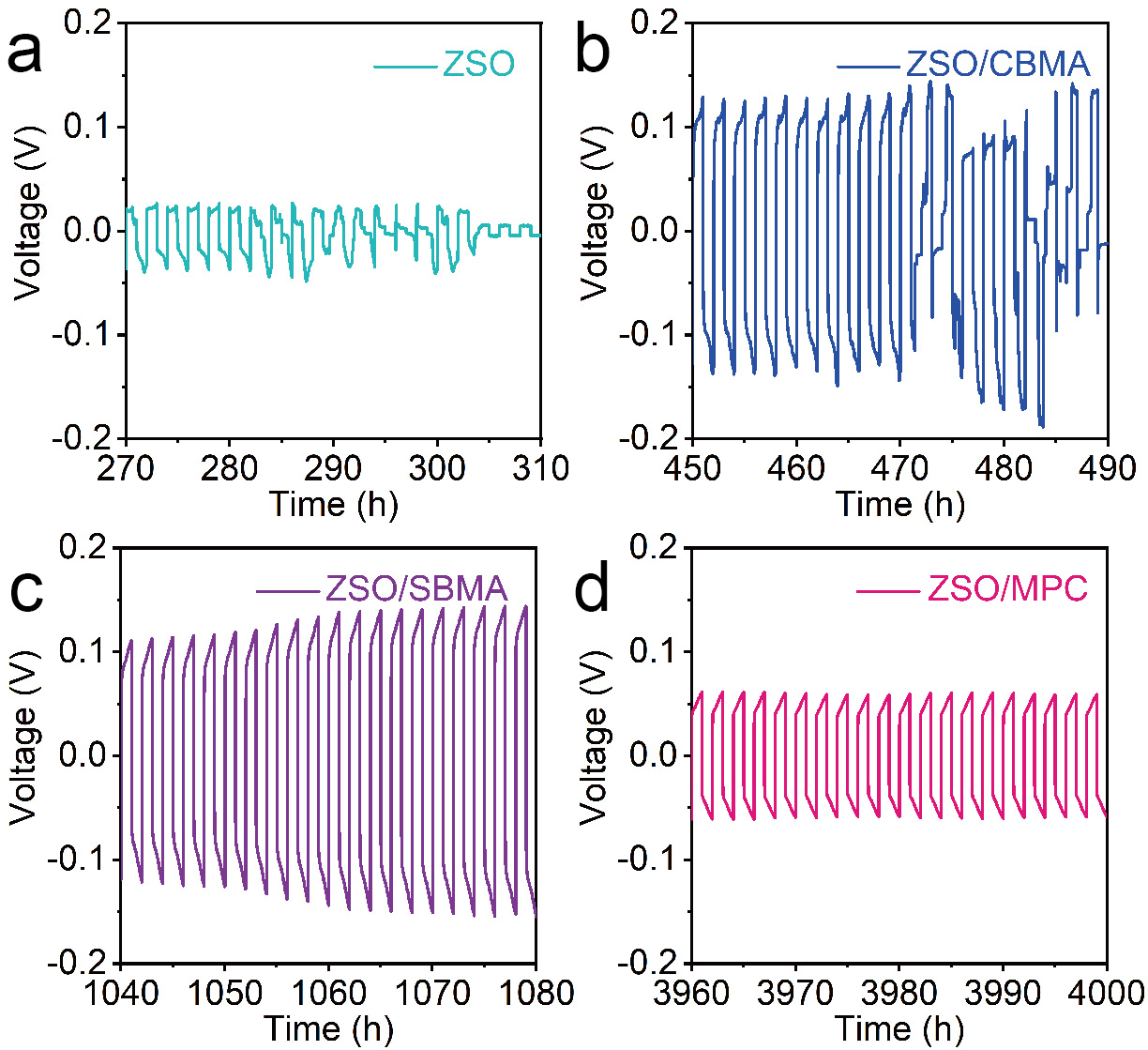


**Fig. S7** Detailed voltage profiles at 1 mA cm^-2^ and 1 mAh cm^-2^


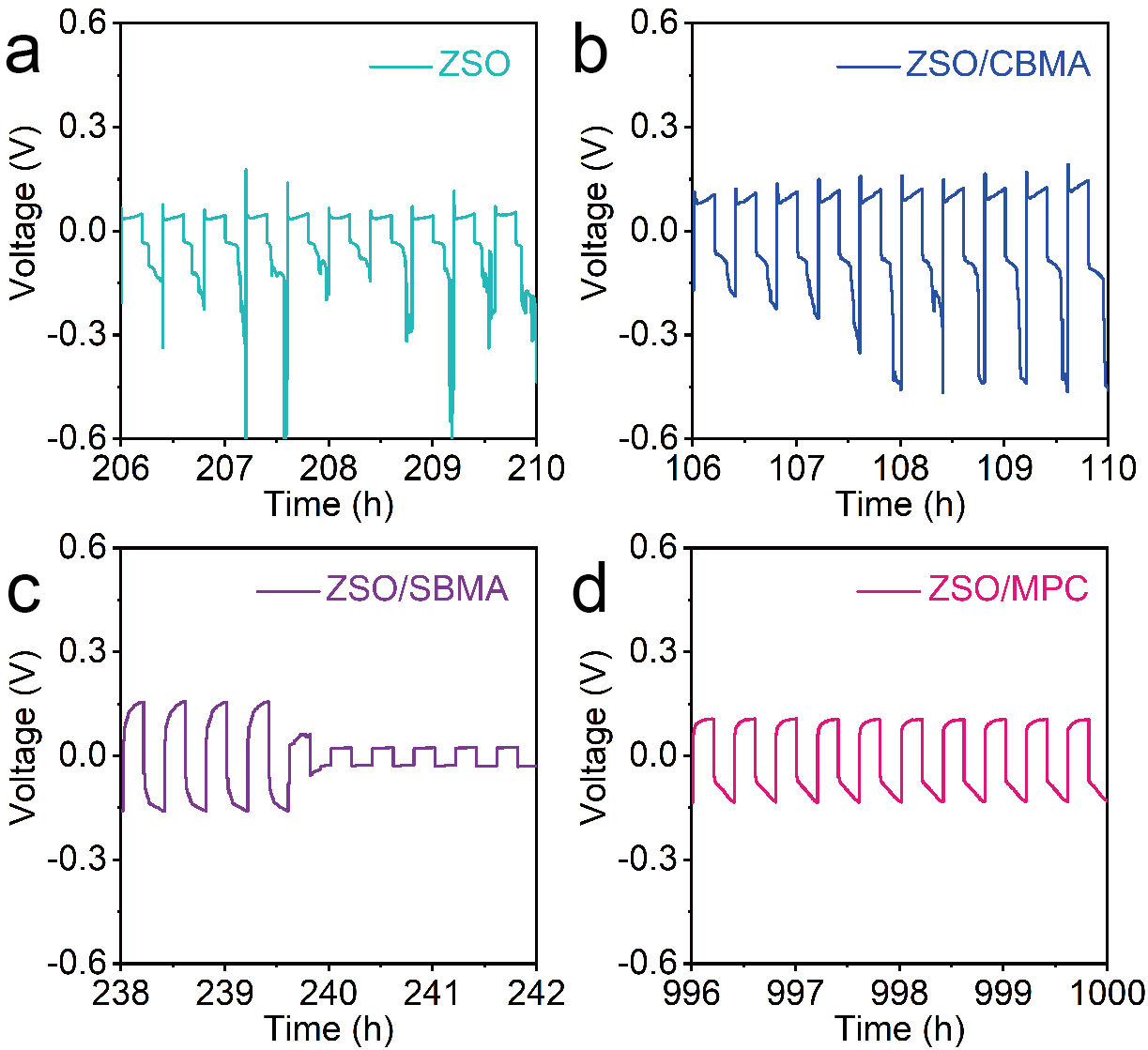


**Fig. S8** Detailed voltage profiles at 5 mA cm^-2^ and 1 mAh cm^-2^


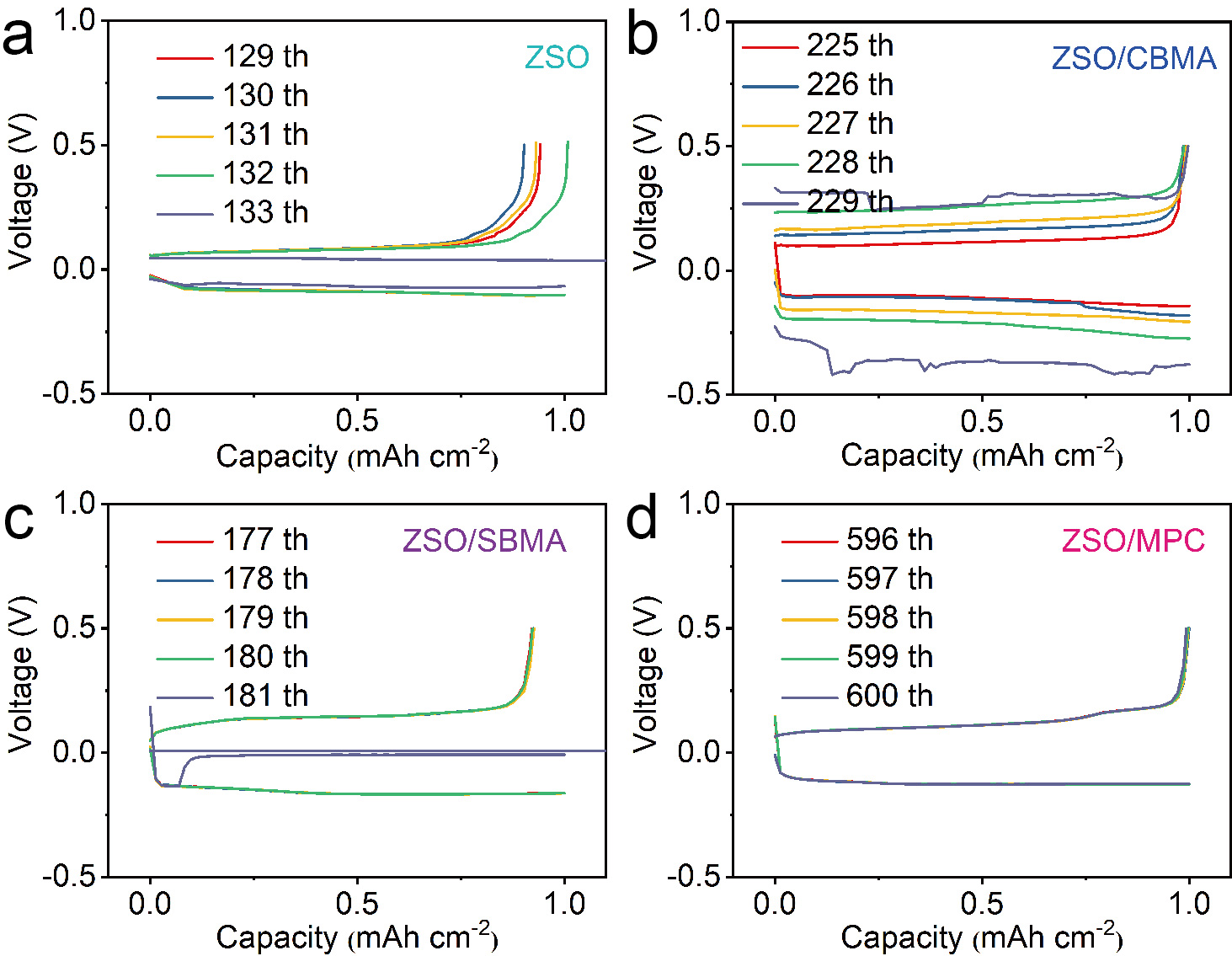


**Fig. S9** Voltage-capacity curves of Zn//Cu cells at 5 mA cm^-2^ and 1 mAh cm^-2^


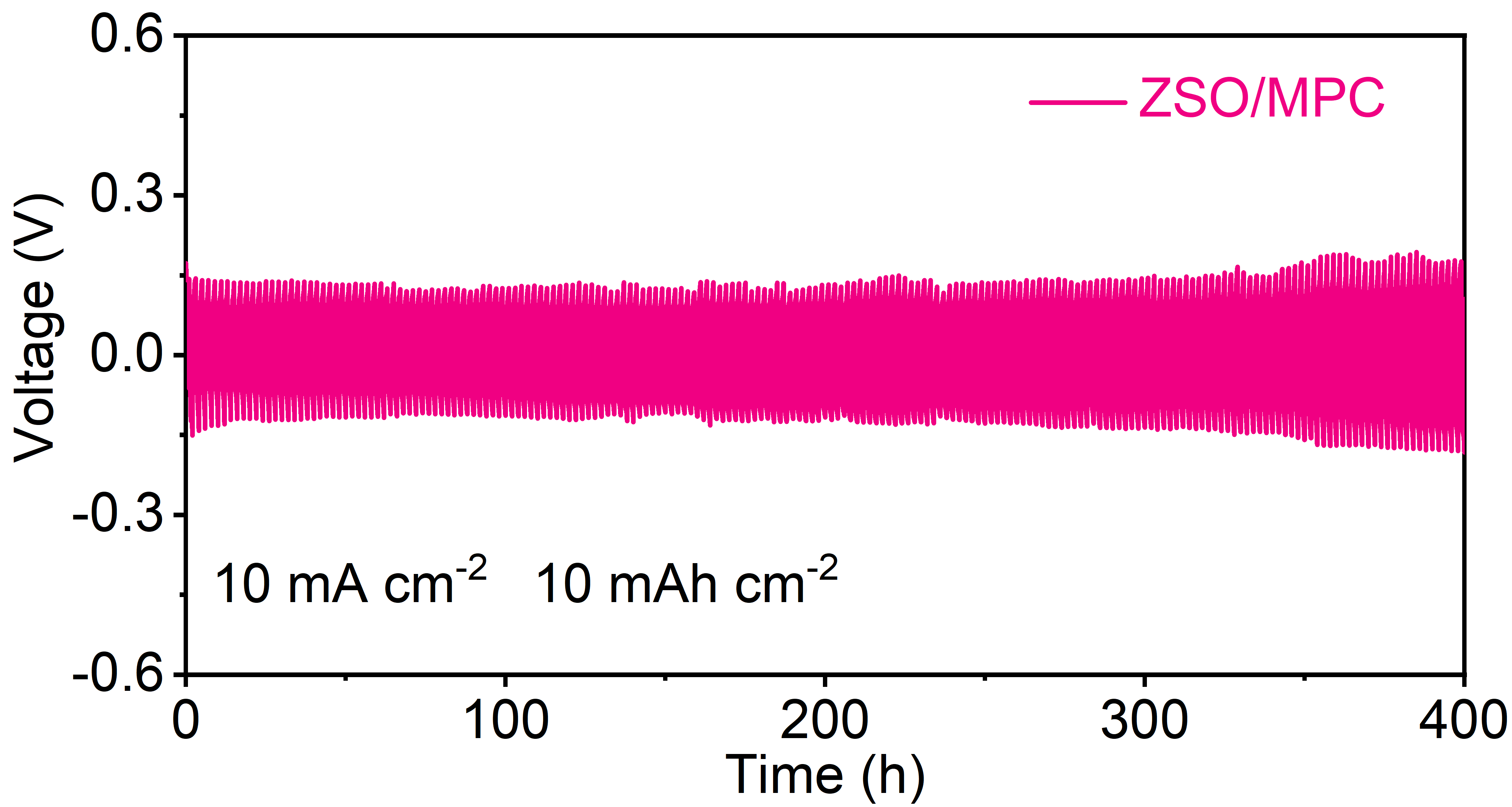


**Fig. S10** Cycling performance of Zn//Zn cell at 10 mA cm^-2^ and 10 mA cm^-2^


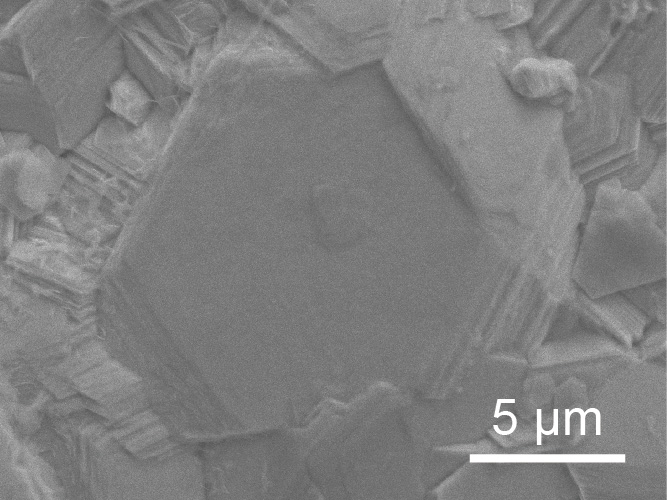


**Fig. S11** High-resolution SEM image of Zn anode in ZSO/MPC cell after cycling.


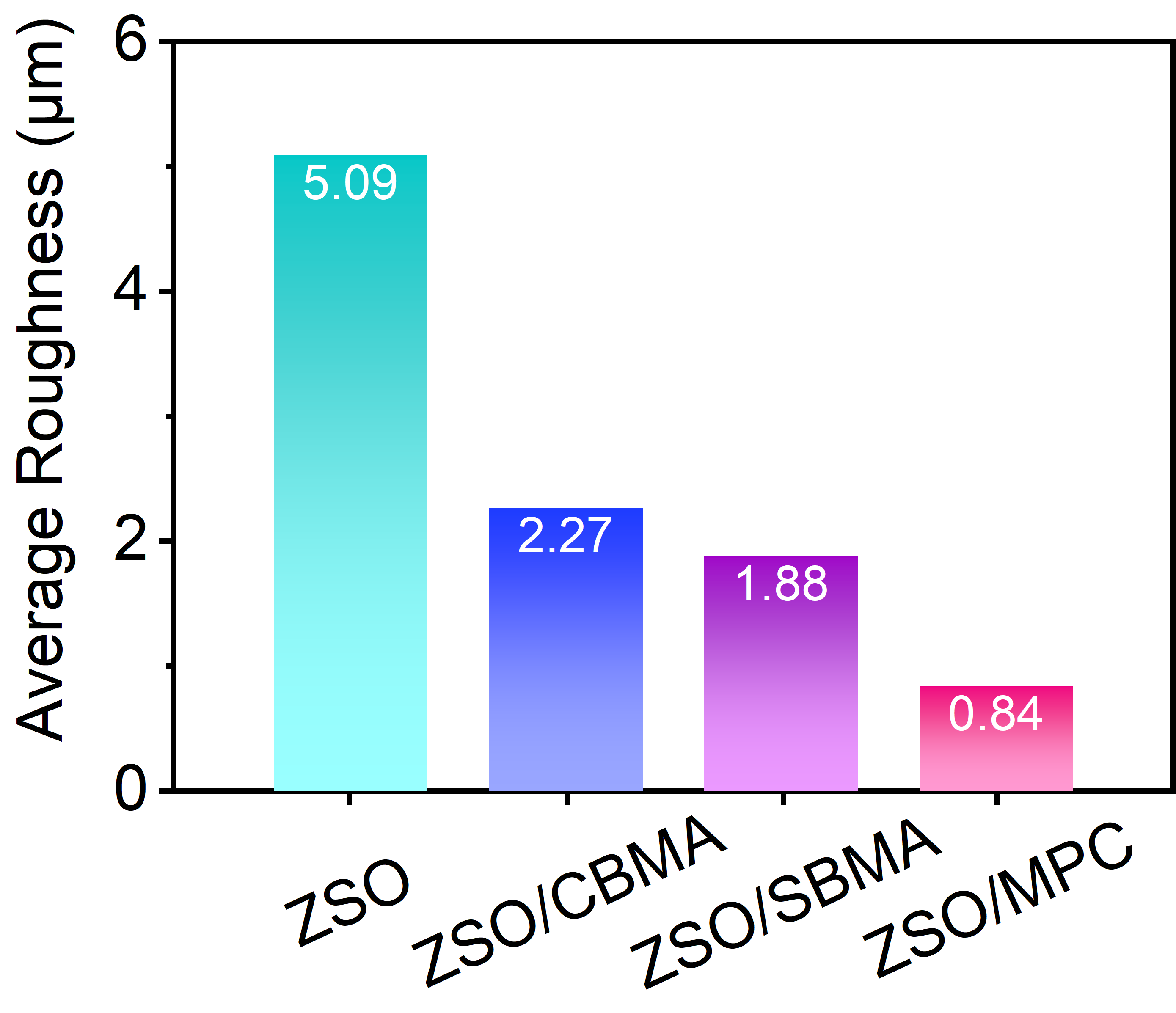


**Fig. S12** Average roughness of cycled Zn with different electrolytes


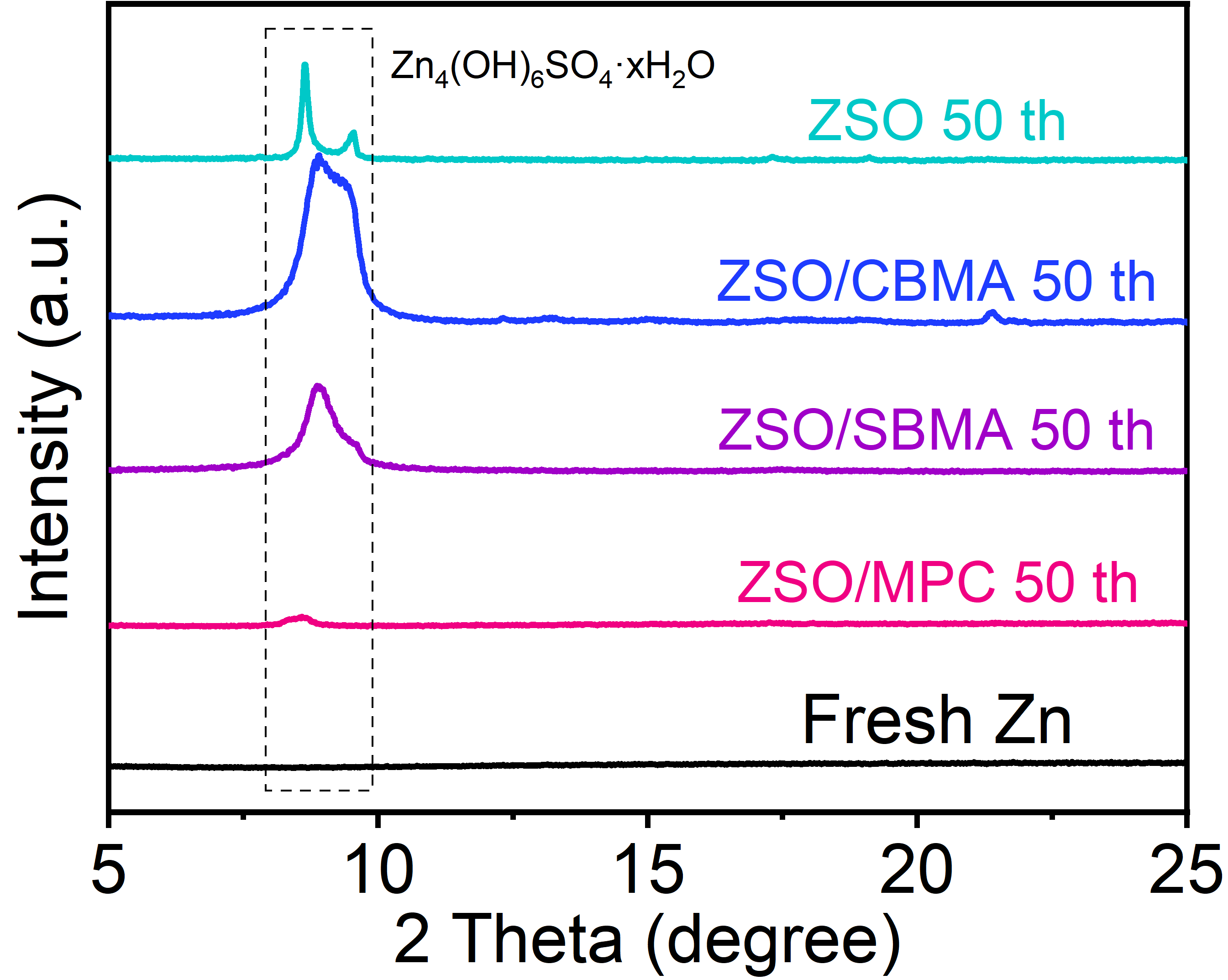


**Fig. S13** XRD patterns of Zn anodes after 50 cycles


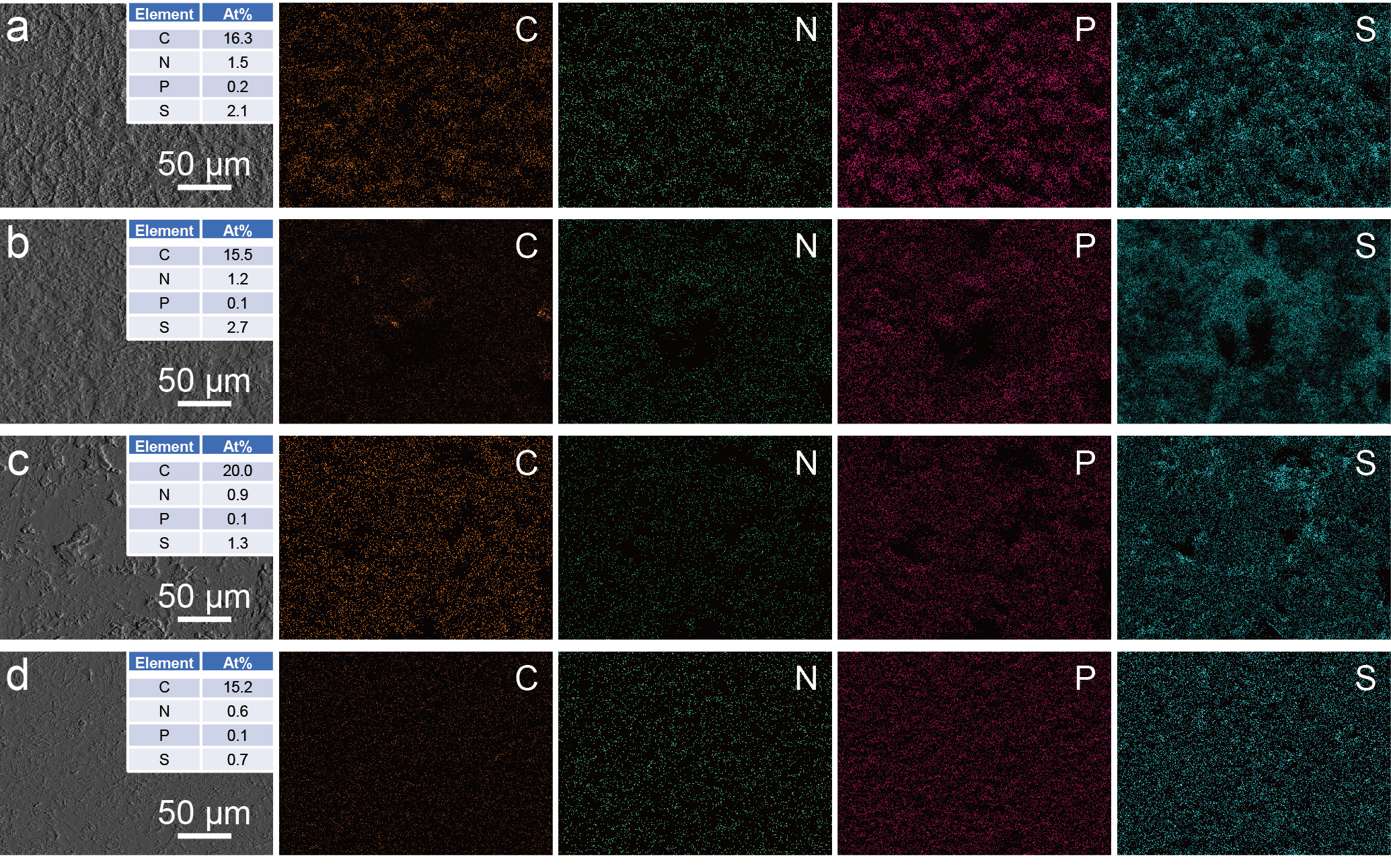


**Fig. S14** SEM image and corresponding element mappings of cycled Zn based on **a** ZSO, **b** ZSO/CBMA, **c** ZSO/SBMA, and **d** ZSO/MPC. The inset is the atomic percentages of C, N, P, and S elements


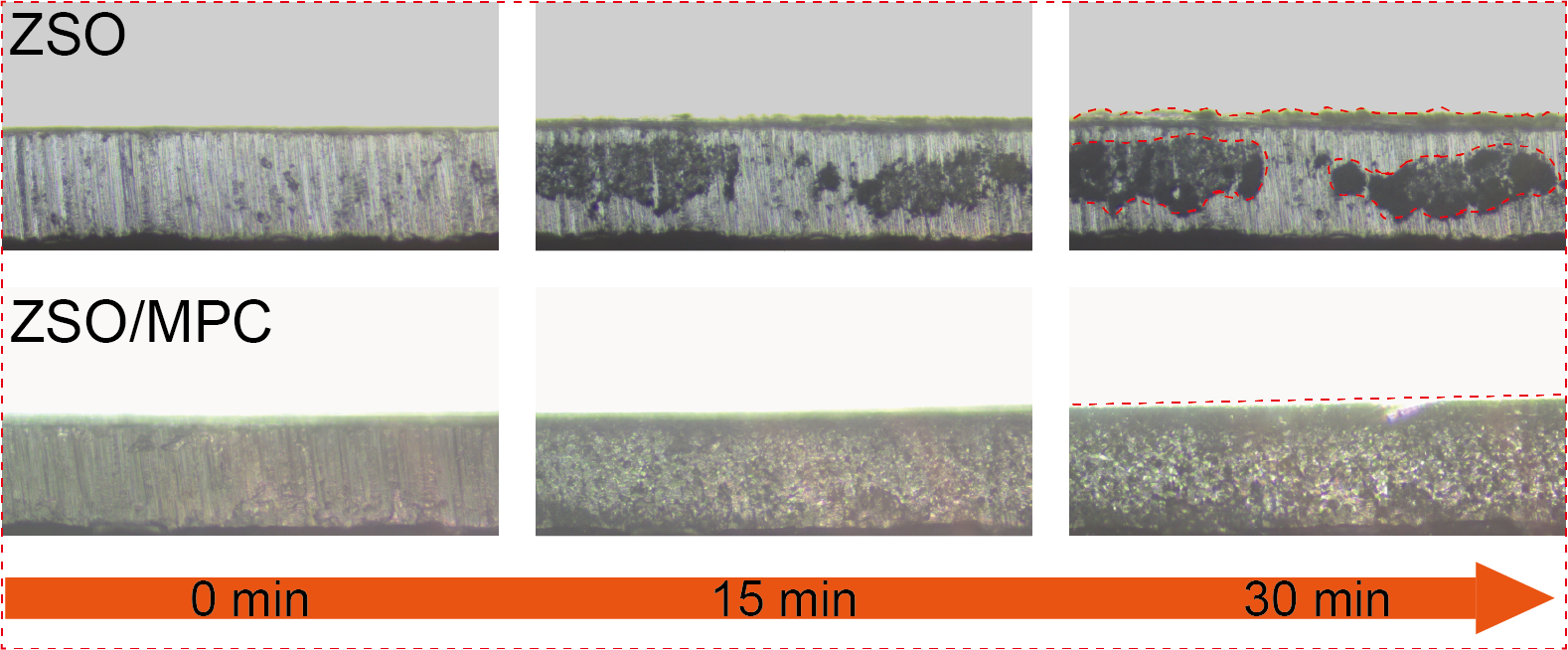


**Fig. S15** *In-situ* optical visualization observations of Zn deposition process


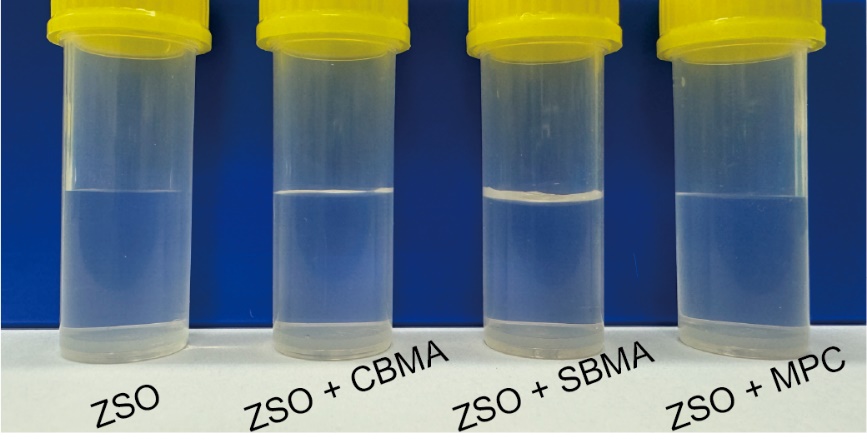


**Fig. S16** Digital photos of different electrolytes


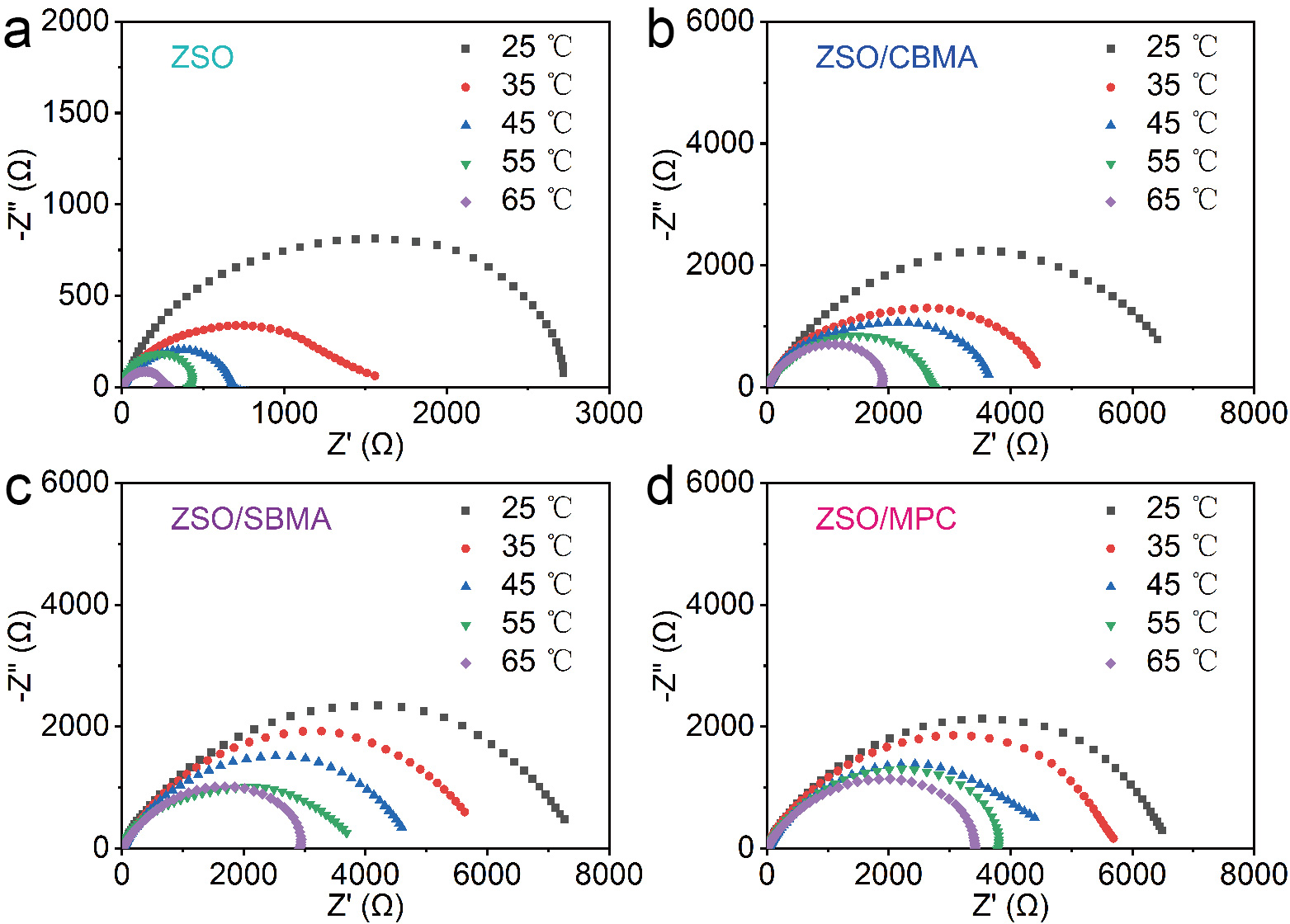


**Fig. S17** Nyquist plots of the Zn//Zn cells at various temperatures


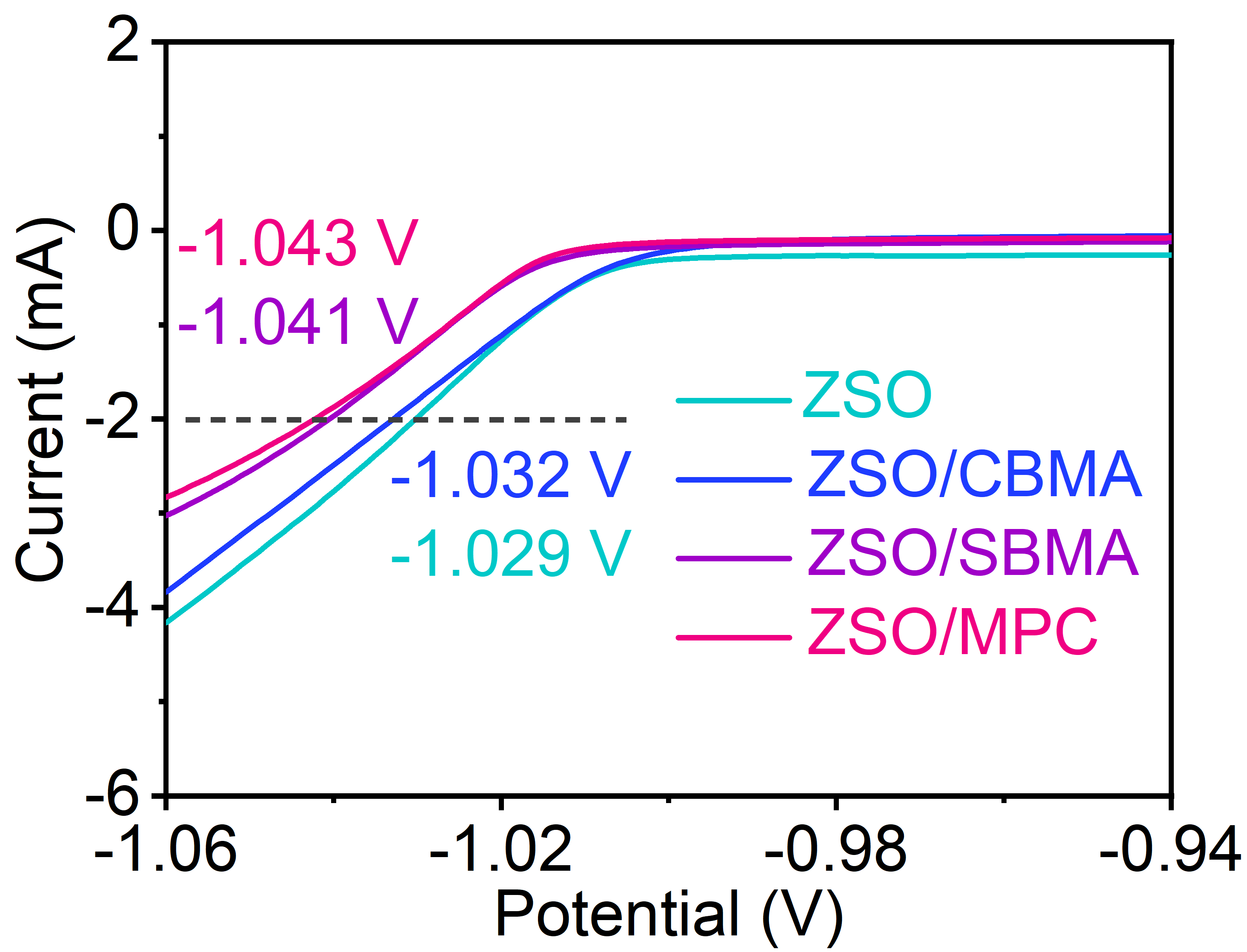


**Fig. S18** LSV curves of different electrolytes


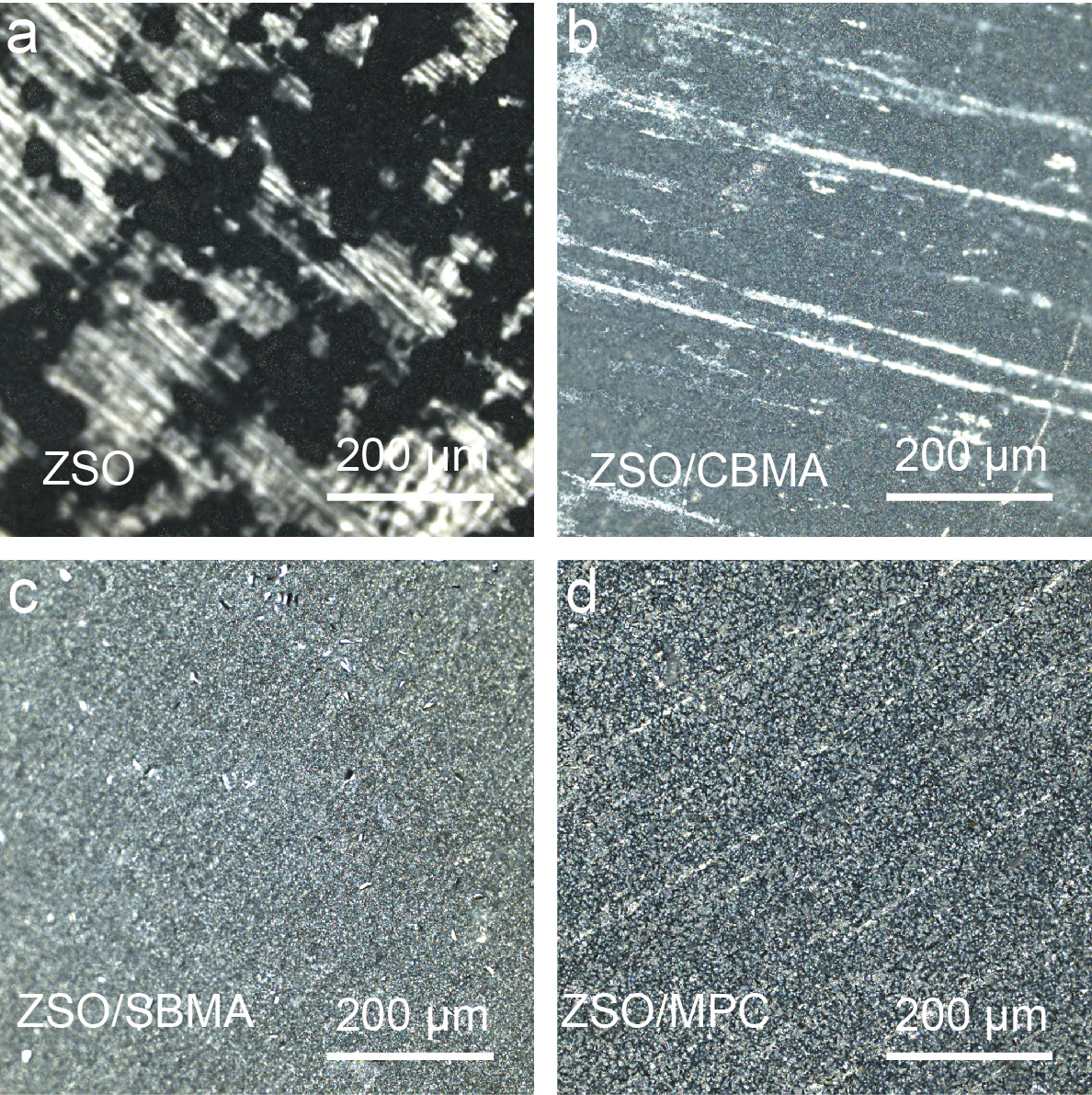


**Fig. S19** CLSM optical images of Zn foils after plating with Zn


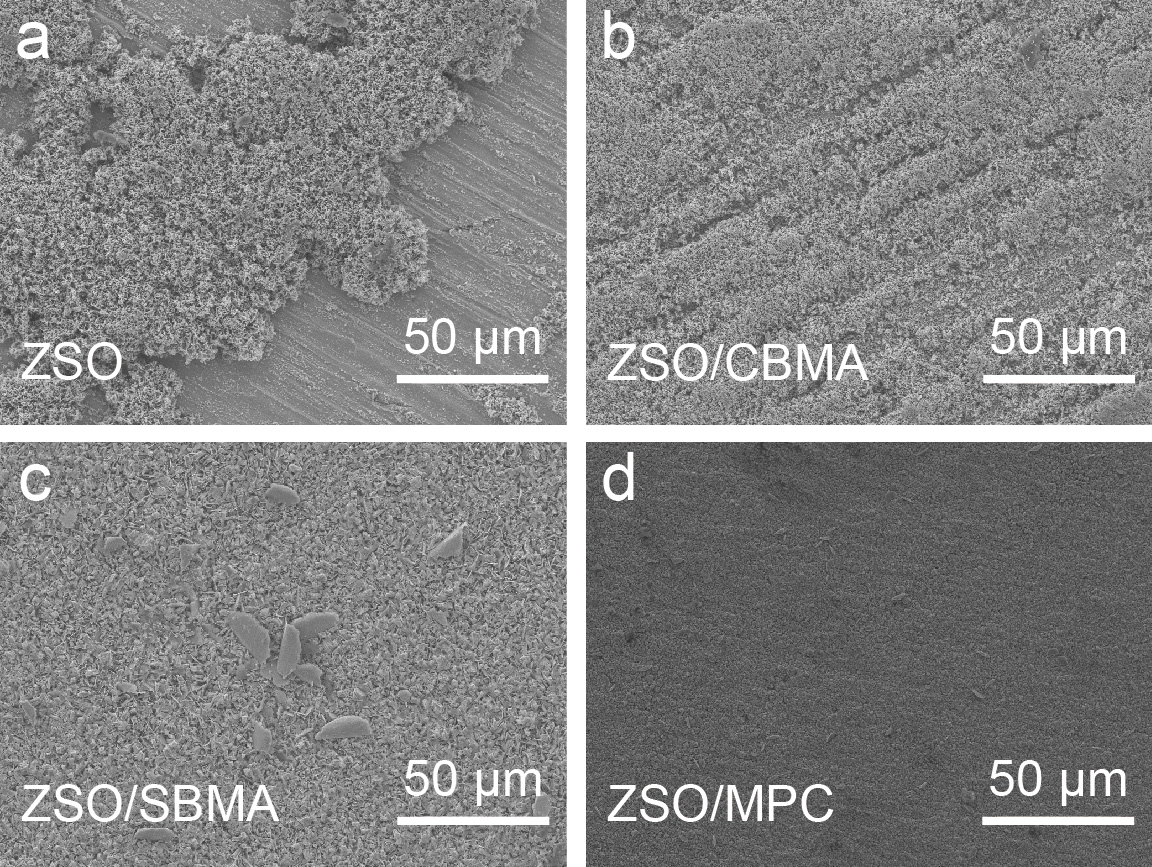


**Fig. S20** SEM images of Zn foils after plating with Zn


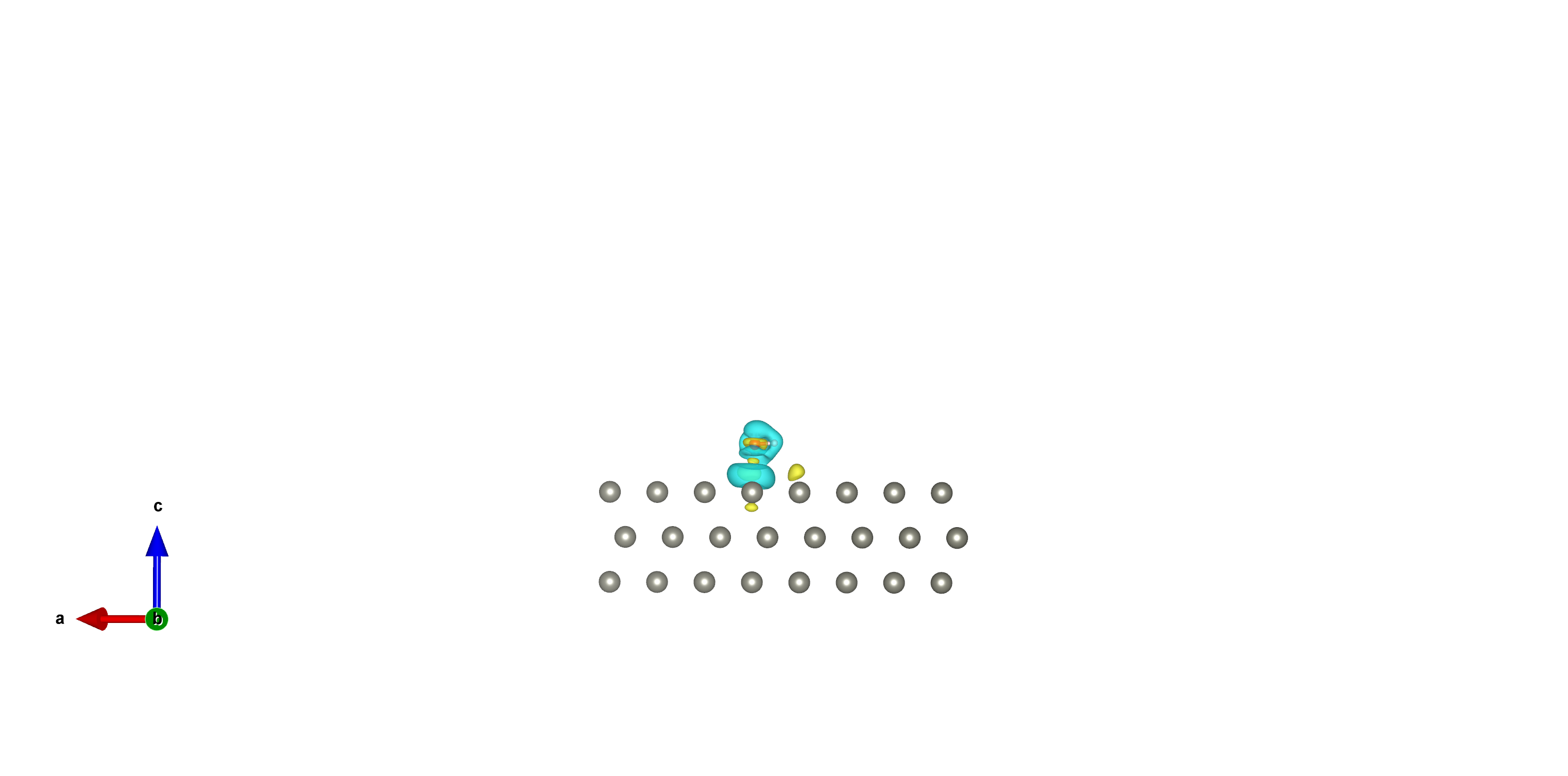


**Fig. S21** The 3D contour mapping differential charge density of H_2_O on Zn (002) plane


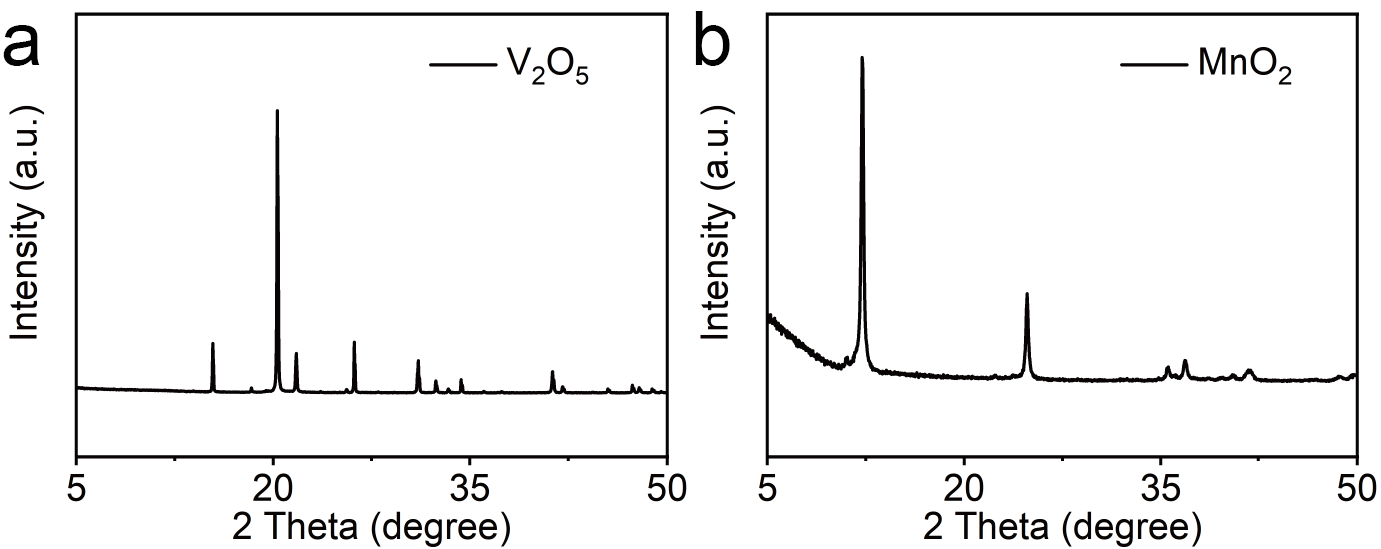


**Fig. S22** XRD patterns of **a** V_2_O_5_ and **b** MnO_2_


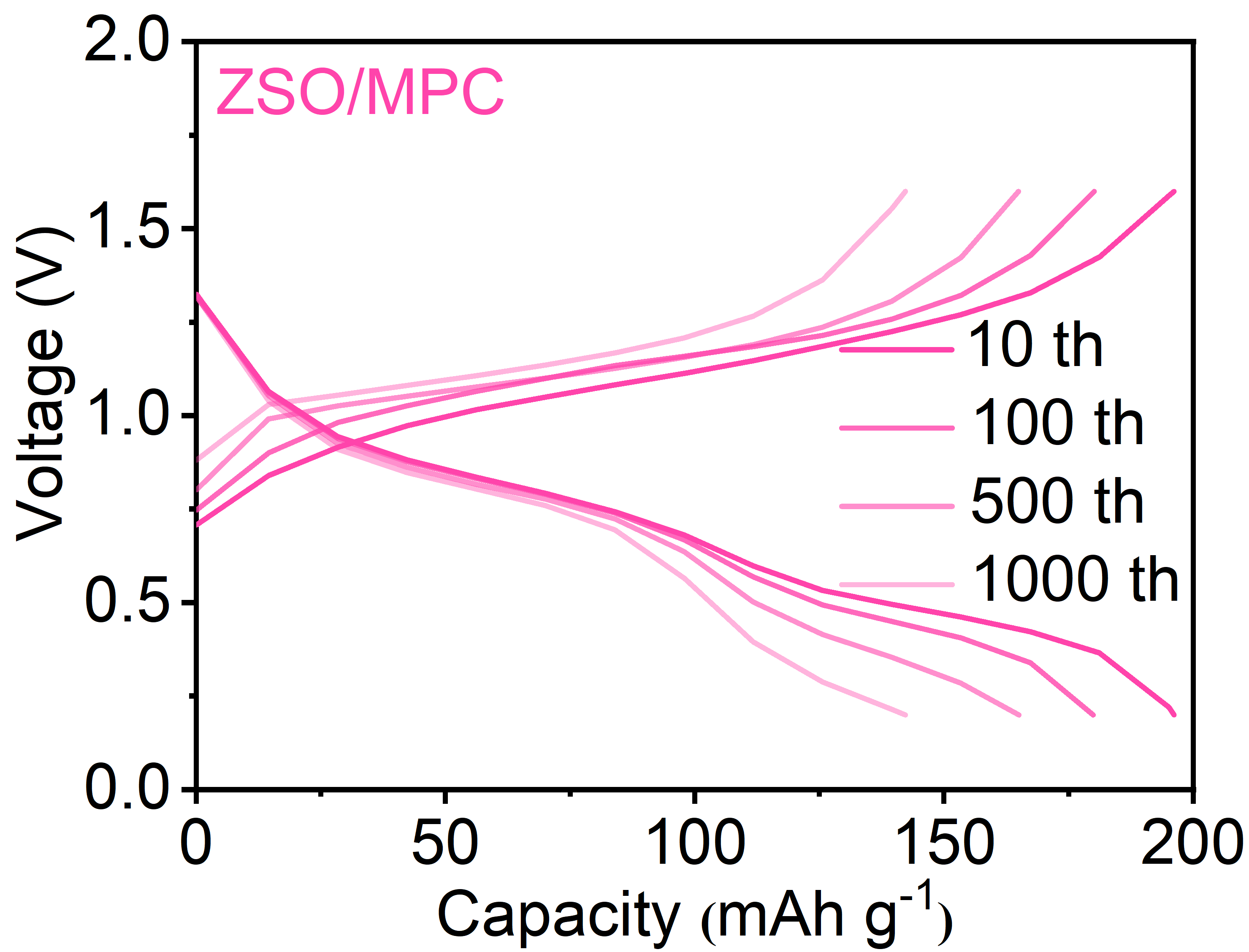


**Fig. S23** GCD profiles of Zn//V_2_O_5_ cell with ZSO/MPC at 5 A g^-1^

**
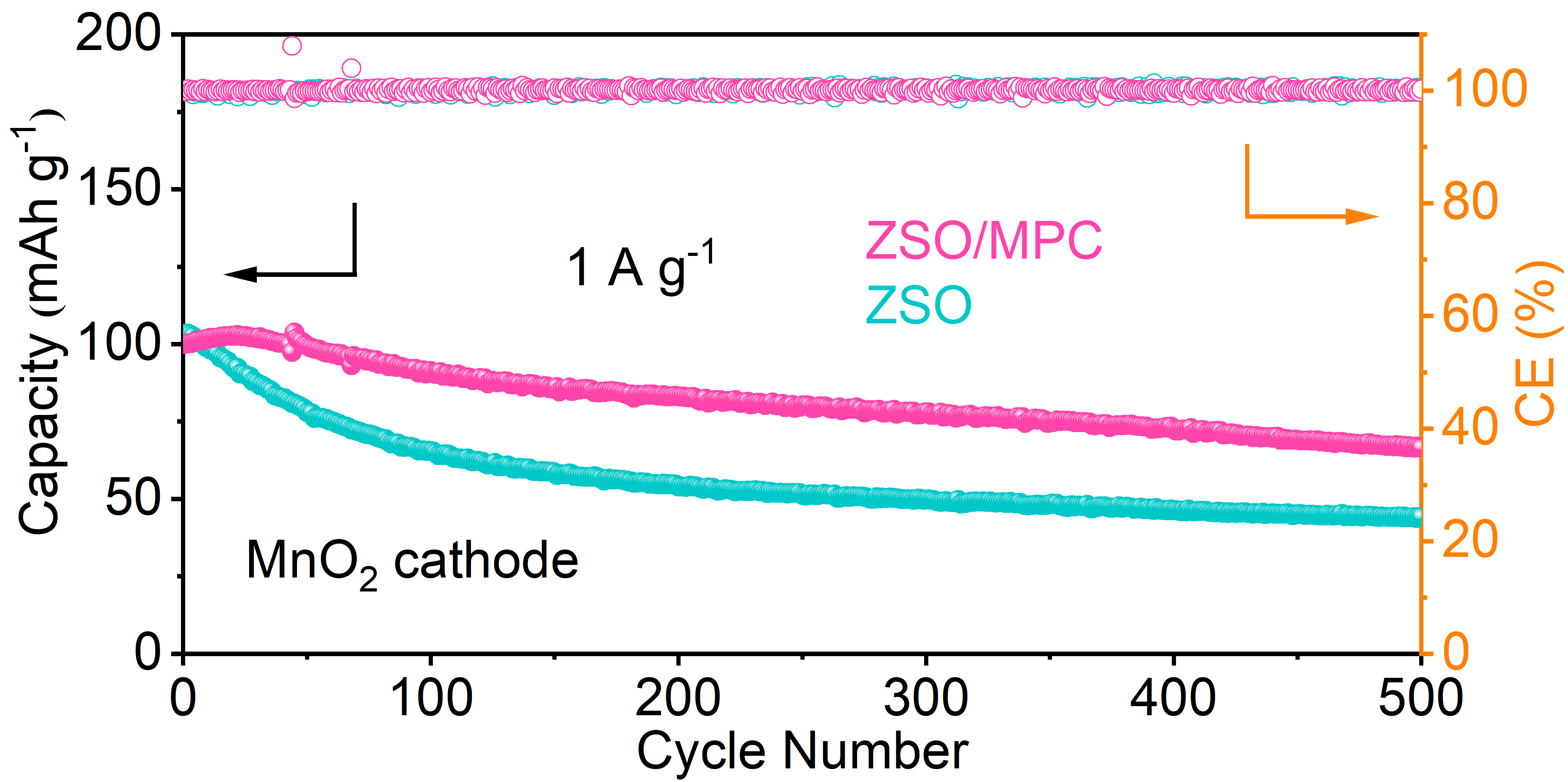
**

**Fig. S24** Cycling performance of Zn//MnO_2_ cell at 1 A g^-1^


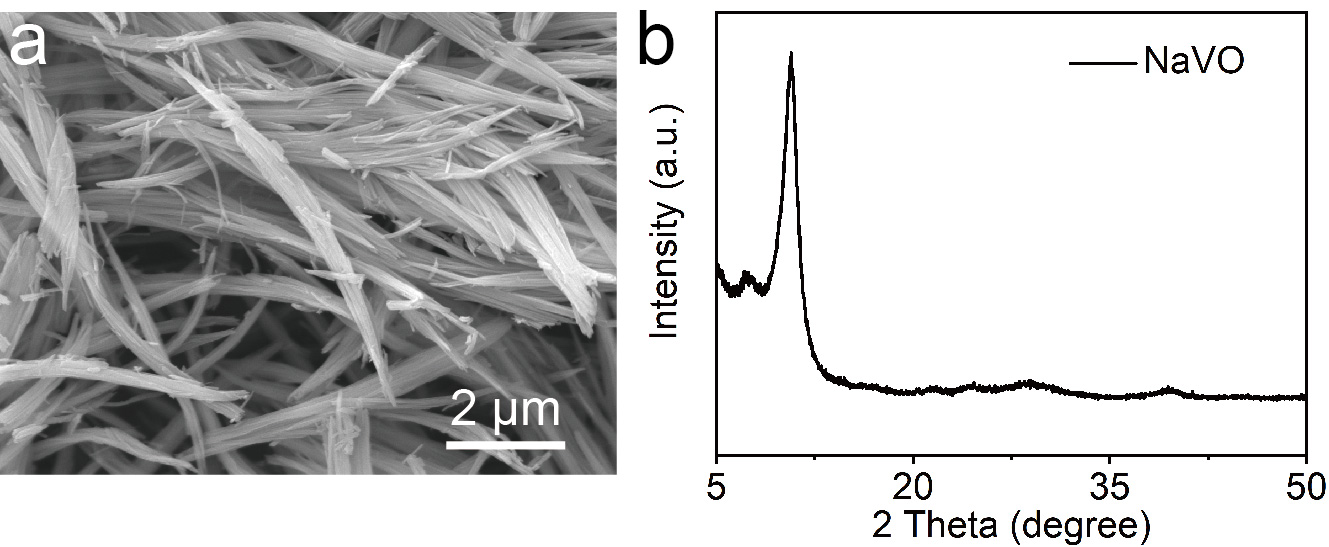


**Fig. S25 a** SEM image and **b** XRD pattern of NaVO

**Table S1** Comparison of electrochemical performance of our work with various works

| Sample | Current density  (mA cm^-2^) | Capacity  (mAh cm^-2^) | Cycle number  (n) | Refs. |
| --- | --- | --- | --- | --- |
| MPC | 1  20 | 1  1 | 2500  8000 | This work |
| GA | 1 | 1 | 1365 | [S1] |
| SDE | 1 | 1 | 1750 | [S2] |
| PGA | 2 | 1 | 800 | [S3] |
| CP | 1 | 1 | 500 | [S4] |
| MES | 0.5 | 0.5 | 800 | [S5] |
| Z10 | 5 | 1 | 1500 | [S6] |
| DA | 10 | 1 | 3500 | [S7] |
| MEP·Br | 5 | 1 | 375 | [S8] |
| FI | 5 | 0.5 | 3000 | [S9] |

**Supplementary References**

1. H. Zheng, Y. Huang, J. Xiao, W. Zeng, X. Li et al., Multi-protection of zinc anode *via* employing a natural additive in aqueous zinc ion batteries. Chem. Eng. J. **468**, 143834 (2023). <https://doi.org/10.1016/j.cej.2023.143834>
2. T. Yan, B. Wu, S. Liu, M. Tao, J. Liang et al., Sieving-type electric double layer with hydrogen bond interlocking to stable zinc metal anode. Angew. Chem. Int. Ed. **63**(47), e202411470 (2024). <https://doi.org/10.1002/anie.202411470>
3. C. Huang, J. Mao, S. Li, W. Zhang, X. Wang et al., Amphoteric polymer strategy with buffer-adsorption mechanism for long-life aqueous zinc ion batteries. Adv. Funct. Mater. **34**(26), 2315855 (2024). <https://doi.org/10.1002/adfm.202315855>
4. Q. Yan, Z. Hu, Z. Liu, F. Wu, Y. Zhao et al., Synergistic interaction between amphiphilic ion additive groups for stable long-life zinc ion batteries. Energy Storage Mater. **67**, 103299 (2024). <https://doi.org/10.1016/j.ensm.2024.103299>
5. X. Liu, B. Xu, J. Lu, J. Han, S. Deng et al., A multifunctional zwitterion electrolyte additive for highly reversible zinc metal anode. Small **20**(12), 2307557 (2024). <https://doi.org/10.1002/smll.202307557>
6. L. Tao, K. Guan, R. Yang, Z. Guo, L. Wang et al., Dual-protected zinc anodes for long-life aqueous zinc ion battery with bifunctional interface constructed by zwitterionic surfactants. Energy Storage Mater. **63**, 102981 (2023). <https://doi.org/10.1016/j.ensm.2023.102981>
7. Y. Yang, Y. Li, Q. Zhu, B. Xu, Optimal molecular configuration of electrolyte additives enabling stabilization of zinc anodes. Adv. Funct. Mater. **34**(32), 2316371 (2024). <https://doi.org/10.1002/adfm.202316371>
8. S. Wang, T. Li, Y. Yin, N. Chang, H. Zhang et al., High-energy-density aqueous zinc-based hybrid supercapacitor-battery with uniform zinc deposition achieved by multifunctional decoupled additive. Nano Energy **96**, 107120 (2022). <https://doi.org/10.1016/j.nanoen.2022.107120>
9. J. Zhu, W. Deng, N. Yang, X. Xu, C. Huang et al., Biomolecular regulation of zinc deposition to achieve ultra-long life and high-rate Zn metal anodes. Small **18**(29), 2202509 (2022). <https://doi.org/10.1002/smll.202202509>
